# Blockade of STING activation alleviates microglial dysfunction and a broad spectrum of Alzheimer’s disease pathologies

**DOI:** 10.1038/s12276-024-01295-y

**Published:** 2024-09-02

**Authors:** Sunwoo Chung, June-Hyun Jeong, Jong-Chan Park, Jong Won Han, Yeajina Lee, Jong-Il Kim, Inhee Mook-Jung

**Affiliations:** 1 Convergence Dementia Research Center, College of Medicine, Seoul National University, 03080 Seoul, Korea; 2 Department of Biochemistry and Biomedical Sciences, College of Medicine, Seoul National University, 03080 Seoul, Korea; 3 Department of Biophysics & Institute of Quantum Biophysics, Sungkyunkwan University, 16419 Gyeonggi-do, Korea; 4 Genomic Medicine Institute, Medical Research Center, Seoul National University, 03080 Seoul, Korea

**Keywords:** Neuroimmunology, Alzheimer's disease

## Abstract

Abnormal glial activation promotes neurodegeneration in Alzheimer’s disease (AD), the most common cause of dementia. Stimulation of the cGAS-STING pathway induces microglial dysfunction and sterile inflammation, which exacerbates AD. We showed that inhibiting STING activation can control microglia and ameliorate a wide spectrum of AD symptoms. The cGAS-STING pathway is required for the detection of ectopic DNA and the subsequent immune response. Amyloid-β (Aβ) and tau induce mitochondrial stress, which causes DNA to be released into the cytoplasm of microglia. cGAS and STING are highly expressed in Aβ plaque-associated microglia, and neuronal STING is upregulated in the brains of AD model animals. The presence of the *APOE* ε4 allele, an AD risk factor, also upregulated both proteins. STING activation was necessary for microglial NLRP3 activation, proinflammatory responses, and type-I-interferon responses. Pharmacological STING inhibition reduced a wide range of AD pathogenic features in App^NL-G-F^/hTau double-knock-in mice. An unanticipated transcriptome shift in microglia reduced gliosis and cerebral inflammation. Significant reductions in the Aβ load, tau phosphorylation, and microglial synapse engulfment prevented memory loss. To summarize, our study describes the pathogenic mechanism of STING activation as well as its potential as a therapeutic target in AD.

## STING inhibition mitigates neurodegeneration in Alzheimer’s disease

In illnesses like Alzheimer’s that cause brain deterioration, the brain’s defense cells, known as microglia, overreact due to harmful proteins, causing brain damage and memory loss. This research aimed to understand how microglia change in Alzheimer’s and find ways to stop their damaging effects. Using mice with Alzheimer’s, they checked if blocking specific immune pathway could fix microglia dysfunction and Alzheimer’s disease pathologies. They discovered that blocking STING, a crucial part of this pathway, reduced microglia dysfunction brain inflammation, decreased the buildup of Alzheimer’s-related proteins, and improved memory in mice. By blocking the STING activation, the study showed a decrease in damaging brain inflammation and improvements in memory function, suggesting a promising strategy for treating Alzheimer’s. Researchers conclude that targeting the STING could offer a new way to fight Alzheimer’s by reducing inflammation and protecting brain health.

This summary was initially drafted using artificial intelligence, then revised and fact-checked by the author.

## Introduction

Abnormal immune activation is a consistent feature of neurodegenerative diseases, including Alzheimer’s disease (AD). The accumulation of Aβ and tau triggers an inflammatory response in reactive glial cells, which disrupts normal synaptic function, damages brain tissue, and eventually causes memory loss^1^. Microglia are innate immune cells that are highly sensitive to surrounding microenvironmental stimuli and thus show a wide range of phenotypic alterations in the degenerating brain^2^. Identifying the causes and key drivers of microglial phenotypic changes is crucial for understanding and modulating the pathophysiology of neurodegenerative diseases^3^. However, due to the wide variety of stimuli present in the diseased brain environment of AD patients, it is challenging to determine which are effective targets for inhibiting microglial dysfunction.

Living organisms spontaneously face internal and external challenges to maintain genomic integrity. The relaxation of heterochromatin^4^, weakening of the nuclear membrane^5^ and accumulation of DNA damage pose internal risks during aging. Exogenous pathogens also pose risks. Both these intrinsic and extrinsic factors are associated with AD. The accumulation of somatic DNA damage and *APP* gene recombination have been reported in the brains of AD patients^6^. Heterochromatin relaxation, which is normally repressed by the polycomb complex, has been reported in sporadic AD neurons^7^. Tau is also known to activate transposable elements^8^. In the inflammatory environment of the AD brain, Aβ induces DNA damage and mitochondrial stress^9^. Moreover, other studies have noted the antimicrobial peptide-like properties of Aβ, suggesting that external factors such as viral pathogens can contribute to AD pathogenesis by triggering the body’s antimicrobial response^10,11^. In searching for a common denominator among these diverse factors, we focused on the immune response against ectopic DNA and specifically the cyclic GMP-AMP synthase (cGAS)-stimulator of interferon genes (STING) pathway.

In mammalian cells, ectopic DNA activates the cGAS-STING pathway to trigger the innate immune response. Double-stranded nucleic acids longer than 45 base pairs activate cGAS to produce cyclic 2’-3’-GMP-AMP (cGAMP), the physiological ligand of STING^12^. STING is activated by a variety of cyclic dinucleotides and induces noncanonical autophagy and downstream immune signaling. Tank-binding kinase 1 (TBK1), which is directly recruited by STING, sequentially phosphorylates interferon-regulatory factor 3 (IRF3) and nuclear factor κB (NF-κB) to trigger the expression of type I interferon (IFN), IFNβ, and inflammatory cytokines, including TNF-α and CXCL10. Autocrine and paracrine type-I-IFN responses foster tissue resistance to viral infection. This process is mediated by IFNAR1/2 and Janus kinase 1 (JAK1)- signal transducer and activator of transcription 1 (STAT1) signaling. The cGAS-STING pathway is known to protect organisms from microbial threats by triggering a broad spectrum of immune responses^13^. Notably, cGAS recognizes nucleic acids in a sequence-nonspecific manner and can thus be adversely activated by endogenous nuclear and mitochondrial DNA (mtDNA) under certain conditions^4,12^. The pivotal roles of the cGAS-STING pathway in the DNA damage response, senescence, antiviral response, and cancer have been highlighted^14^. However, increasing evidence implicates dysregulation of the cGAS-STING pathway in neurological disorders, including multiple sclerosis^15^, Parkinson’s disease^16^, amyotrophic lateral sclerosis (ALS)^17^, Huntington’s disease^18^, and recently, amyloidosis^19^ and tauopathy^20^.

Here, we showed that STING inhibition can be an effective microglia-modulating therapy for AD. The cGAS-STING pathway can be activated by Aβ, tau, and *APOE* ε4. Both neuronal and microglial STING were upregulated in the brains of AD model mice. Proinflammatory microglial polarization, the IFN response, and NLRP3 activation were dependent on STING activation. In App^NL-G-F^/hTau-double knock-in mice, pharmacological STING inhibition mitigated brain inflammation and microglial synapse engulfment. Finally, the Aβ burden, tau phosphorylation, and cognitive impairment were significantly ameliorated by STING inhibition. Taken together, our study showed the therapeutic potential of STING inhibition in AD.

## Materials and methods

### Mouse model

All animal studies were conducted under the approval of the Seoul National University Institutional Animal Care and Use Committee. App^NL-G-F^ knock-in (KI) mice and App^NL-G-F^/hTau-double KI (dKI) mice on the C57BL/6 (B6) background were used for the experiments. This mouse model generates humanized Aβ peptides and 6 isoforms of human tau without causing protein overexpression or autophagic deficit^21^. An in-house AD-like pathology (ADLP) mouse model was established as previously described^22^. Moreover, ADLP^APP/PS1^ and ADLP^APT^ mice have a *PSEN1* mutation that genetically disrupts autophagic flux^23^. Since the STING protein lifecycle is closely related to autophagy, dKI mice were mainly used in this study. Female mice were used due to their early appearance of AD pathology and rapid progression. Pregnant wild-type ICR mice were used to obtain pups for primary culture.

### Cultured microglia stimulation

Aβ (2 μM) and the wild-type human tau peptide Tau441 (rPeptide, 25 nM) were diluted in FBS-free DMEM for microglial stimulation. An equal amount of DMSO was used as a control. Cells were pretreated with STING inhibitors for 1 h before stimulation. cGAMP (InvivoGen) was directly diluted in DMEM to avoid adverse effects on microglial activation by Lipofectamine. Bafilomycin A1 (Sigma) was used to block lysosomal flux in cGAMP-treated cells.

### Pharmacological STING inhibition in App^NL-G-F^/hTau-dKI mice

Ten-month-old dKI mice were intraperitoneally injected with the STING inhibitor H-151 for 2.5 months. Briefly, 210 μg of H-151, which is equivalent to 7 mg/kg for mean body weight, was disolved in DMSO and diluted in 10% Tween 80 in PBS and injected 3 times a week; this protocol was modified from a previous study^17^.

### SDS‒PAGE and western blotting

Cells were rinsed with PBS and lysed with RIPA buffer containing PMSF, PI and PPI-I and PPI-II. Brain tissue homogenates aliquoted for protein analysis were mixed with 5x RIPA buffer for complete lysis. The total protein concentration was measured by a BCA assay, normalized, and denatured by Nu-PAGE 4x sampling buffer. The samples were separated by electrophoresis on 4–12% Bis-Tris Nu-PAGE gradient gels (Invitrogen). Proteins were transferred to PVDF membranes (Millipore) and blocked with 5% skim milk in TBST (TBS containing 0.05% Tween-20). Primary antibodies, including 3% bovine horse serum (BSA) and 0.05% NaN3, were diluted in TBST and detected by using appropriate HRP-conjugated secondary antibodies. All primary antibodies were used at a 1:1000 dilution, with the exception of β-actin (1:2000).

### Aβ fractionation and enzyme-linked immunosorbent assay (ELISA)

Aβ_40_ and Aβ_42_ ELISA kits (Wako) were used following the manufacturer’s instructions with minor modifications. Briefly, brain homogenates in TBS were centrifuged at 21,000 × *g* for 20 min at 4 °C, and the resulting supernatants were saved as the TBS fraction. Gu-HCl solution was added to the TBS fraction to reach a concentration of 0.5 M before ELISA. The TBS-insoluble pellets were dissolved in a 6 M Gu-HCl solution supplemented with PI, sonicated and vortexed before 1 h of incubation at room temperature. The solubilized pellets were ultracentrifuged at 200,000 x *g* for 20 min at 4 °C, after which the supernatants were diluted 12-fold with TBS and used as the Gu-HCl fraction. The fractions were diluted to bring the Aβ levels within the detection range.

### Measurement of cGAMP levels

cGAMP levels was measured as described in a previous study with modifications^17^. The cortical tissues of App^NL-G-F^ mice were homogenized in PBS and immediately diluted in methanol to 1:5 degrees. The diluted homogenate was sonicated and vortexed for full lysis and centrifuged at 21,000 × *g* for 5 min at 4 °C to remove tissue debris. The supernatants were deep-frozen in LN2, freeze-dried to lyophilize the tissue extracts and then resuspended in PBS before ELISA was performed. The level of cGAMP was measured with a 2’,3’-cGAMP ELISA kit (Cayman Chemical).

### Subcellular fractionation and measurement of cytoplasmic DNA levels

Cytoplasmic, mitochondrial and nuclear fractionation was performed as described in a previous study^24^. Cytoplasmic fraction dsDNA levels were measured with a Quant-iT™ PicoGreen™ dsDNA assay kit (Invitrogen). The fluorescent signals in each group were normalized to the amount of β-actin in the cytoplasmic fraction.

### Brain microglia isolation and transcriptome analysis

After perfusion with ice-cold HBSS, dissected brain tissue pieces were incubated for 30 min in papain-based enzyme solution supplemented with DNaseI. To prevent adverse ex vivo activation, a triple inhibitor cocktail (actinomycin D, anisomycin, and triptolide) was used as described in a previous study^25^. The enzyme solution was removed by centrifugation, and the washed tissue pieces were passed through a 70 μm mesh filter to obtain a cell suspension. The myelin debris was removed by using debris removal solution (130-109-398, Miltenyi Biotec), and the cell pellets were resuspended in MACS buffer (0.5% BSA and 2 mM EDTA in DPBS). Microglia were isolated by using a CD11b+ magnetic microbead (130-093-636, Miltenyi Biotec) following the manufacturer’s instructions. RNA was extracted with an RNeasy PLUS Mini kit (QIAZEN). The RNA was further processed for sequencing by the SNU Genomics Institute. RNA quality was assessed with an RNA 6000 Pico Kit with a Bioanalyzer (Agilent). The cDNA library was prepared with a QuantSeq 3’-mRNA-Seq Library Prep Kit (Lexogen, Inc.). The sample libraries were sequenced using an Illumina HiSeqX system. RNA was mapped to the mouse genome GRCm39, and DEG analysis was performed with the R DESeq2 package. The DEG cutoff was set as baseline ≥ 20, log2FC > |0.8 | , and *p-*value < 0.05. Gene Ontology (GO) analysis was performed with gProfiler. Gene set enrichment analysis (GSEA) was performed with mouse MSigDB gene sets.

### *TMEM173* knockout iPSC line establishment and iMG differentiation

Human iPSC cell lines were maintained as previously described^26^. The *TMEM173* knockout iPSC line was established by utilizing the CRISPR all-in-one nonviral vector system (abm). On Day 0, the vectors were transfected into the *APOE* ε4/ε4 iPSC line using Lipofectamine^TM^ Stem (Thermo Fisher Scientific). On Day 3, the culture medium was replaced with Y-27632-supplemented mTeSR1+ to prevent cell death, and GFP-positive cells were sorted and seeded in Matrigel-coated 96-well plates with a SH800S cell sorter (Sony Biotechnology). Grown colonies were sequentially passaged into 24-, 12-, and 6-well plates. For confirmation, genomic DNA was extracted, and target sites were PCR amplified and sent for sequencing. Insertions/deletions were confirmed with the Benchling platform (https://www.benchling.com/) and the CRISP-ID v1.1 webtool (http://crispid.gbiomed.kuleuven.be). Potential off-target effects were predicted by the CRISPR RGEN Cas-OFFinder webtool (http://www.rgenome.net/cas-offinder/). iMG differentiation was performed as described in a previous study with some modifications^27^. The STING-KO iPSC line was obtained from a *TMEM173* sgRNA: 5’-AGGTACCGGAGAGTGTGCTC-3’-transfected clone.

### Immunofluorescence staining

Cultured cells were washed with PBS and fixed with 4% paraformaldehyde for 12 min. The fixed samples were sequentially incubated with 0.3% Triton X-100 for 10 minutes and with blocking solution (2% horse serum, 0.5% BSA) for 20 min at room temperature. Stored brain sections were washed with PBS, treated with 1% Triton X-100 for 10 min and incubated in blocking solution (5% horse serum, 0.5% BSA, 0.3% Triton X-100 in PBS) for 1 h at room temperature. Antigen retrieval was performed by incubation with 70% formic acid for 20 min before blocking. The samples were then incubated with primary antibodies overnight at 4 °C, incubated with fluorophore-conjugated secondary antibodies for 1 hour in the dark at room temperature, counterstained with DAPI for 10 min and mounted. Primary antibodies were diluted in blocking solution. Methoxy-X04 (R&D Systems, 1:100) was added to the secondary antibody solution. For nucleic acid staining, the sections were incubated with diluted acridine orange (2 µg/ml) or DAPI (1:5000) in PBS. Tissues were imaged using Zeiss LSM 700, LSM980 AiryScan (Carl Zeiss) and BC43 (Oxford Instruments) confocal microscopes. Images were analyzed by ImageJ and IMARIS (bitplane) software.

### Y-maze test

Mice were allowed to freely explore the environment in the middle of the maze for 8 min. Spatial working memory function was measured as the percentage of spontaneous alternation (%), [number of alternations]/[total arm entry number - 2]. The number of alternations was counted when the mouse continuously entered three different arms.

### Novel objective recognition (NOR) test

On Day 0, the mice were allowed to freely explore the environment in the middle of the box for 10 min. On Day 1, the mice were introduced to the familiar objects for 10 min for the familiarization session. On Day 2, one familiar object was replaced with a novel object, and the mice were introduced to the new object for 5 min. The exploratory behavior was recorded. All behavioral experiments were performed in a dark environment.

### Statistical analysis

The quantified data are presented as the mean ± SEM. All the statistical analyses were performed using GraphPad PRISM 8. The data were analyzed with an unpaired Student’s *t-*test and one-way or two-way ANOVA followed by Tukey’s multiple-comparisons test or the Mann‒Whitney test. *P* values equal to or lower than 0.05 were considered to indicate a statistically significant difference.

Certain methods are included in the Supplementary Information. All antibodies used are listed in Supplementary Table 1.

## Results

### The cGAS-STING pathway is activated by diverse AD pathologies and risk factors

**Fig. 1:**
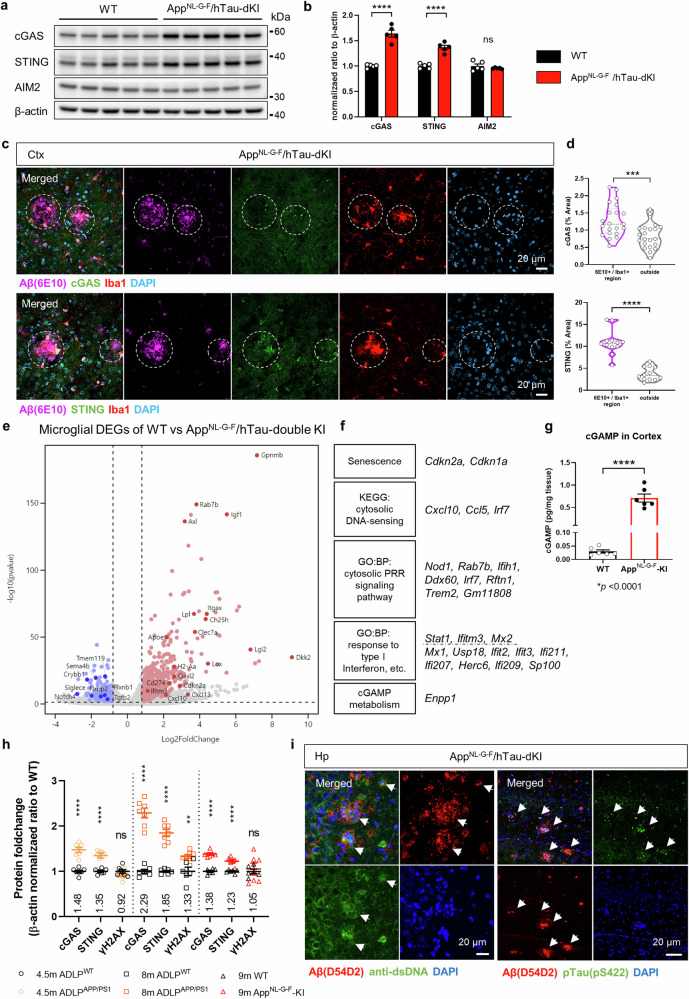
The cGAS-STING pathway is activated in AD model mice. **a** Representative immunoblots of brain cGAS, STING, and AIM2 protein levels in App^NL-G-F^/hTay-dKI (dKI) mice. **b** Quantification of cGAS, STING, and AIM2 total protein levels. **c**, Representative images showing cGAS, STING, Iba1, and Aβ immunostaining in the cortex of 9-month-old dKI mice (*n* = 5,5). The circles indicate the proximal area of the Aβ plaque. Scale bars, 20 μm. **d** Quantification of cGAS and STING signal coverage in the plaque proximal Iba1-positive area and outside of the Iba1-positive area. *n* = 21, *N* = 7 for cGAS; *n* = 12, *N* = 6 for STING. **e** Volcano plot of RNA-seq data for brain-isolated microglia from WT and dKI mice. Red and blue dots represent DEGs with a log2FC > |0.8| and a *p-*value < 0.05. **f** DEGs related to cGAS-STING pathway activation. **g** Measurement of cGAMP levels in the cortex of App^NL-G-F^-KI mice by ELISA. *N* = 6,6. **h** Summary of the quantification of cGAS, STING and γH2AX levels in App^NL-G-F^ and ADLP^APP/PS1^ mouse brains, which revealed that cGAS and STING were induced earlier than γH2AX. Related to Supplementary Fig. 1. *N* = 7.7 for 4.5 m ADLP^APP/PS1^ mice, *N* = 6.7 for 8 m ADLP^APP/PS1 mice^, *N* = 6.6 for App^NL-G-F^ mice. **i** Representative immunostaining images of anti-Aβ, anti-dsDNA, and anti-p-tau antibodies in the brains of dKI mice. The arrows indicate DNA and p-tau signals in the proximal plaque area. Scale bars, 20 μm.In (**b**), (**d**), (**g**), and (**h**), the data are presented as the mean ± standard error the mean (SEM). Statistical significance was determined by Student’s *t-*test. **p < 0.01, ***p < 0.001, ****p < 0.0001. *n* indicates biological replicates, and *N* indicates individual mice.

Microglia-clustered neuritic Aβ plaques are an obvious pathological feature of AD, and reactive microglia induce neurotoxic inflammation. cGAS and STING protein levels were significantly increased in the brains of two independent AD model mice, App^NL-G-F^ and ADLP^APP/PS1^ mice. In contrast, another reported ectopic DNA sensor, AIM2, was not changed (Supplementary Fig. 1a–c). Since AD has both Aβ and tauopathies, we further utilized App^NL-G-F^/hTau-dKI mice and found the same expression patterns (Fig. 1a, b). cGAS and STING were highly expressed in Aβ plaque-clustered microglia (Fig. 1c, d). Microglia in the brains of dKI mice exhibited typical neurodegenerative transcriptomic features, such as downregulation of the homeostatic gene *Tmem119* and upregulation of scavenging receptors (*Trem2*, *Clec7a*, *and Axl*) and genes associated with immune activation (*Itgax, Ccl6, and Cxcl10*) and lipid metabolism (*ApoE and Lpl*) (Fig. 1e). Consistently, the transcript levels of cGAS-STING pathway members were increased in microglia. Senescence is a driving force for cGAS-STING pathway activation^28^. The senescence marker genes (*Cdkn1a*, *Cdkn2a*) and cGAS-STING pathway downstream response genes (*Irf7*, *Stat1*, *Ifitm3*, *Cxcl10*) were upregulated in dKI microglia, and the expression of the cGAMP-degrading enzyme *Enpp1* was also upregulated (Fig. 1f). The App^NL-G-F^ mouse brain cortex contained significantly high levels of cGAMP, a product of cGAS and ligand for STING, confirming pathway activation (Fig. 1g). Although its cellular localization is divergent, cGAS often colocalizes with the DNA damage marker γH2AX under certain stress conditions^29^. Therefore, we compared the increase in γH2AX levels with that in cGAS and STING levels. cGAS and STING were more highly expressed in ADLP^APP/PS1^ mice than in App^NL-G-F^ mice at similar ages, with a pattern similar to that of amyloidosis. *APP* and *PSEN1* mutant gene overexpression caused ADLP^APP/PS1^ mice to develop more aggressive amyloidosis than App^NL-G-F^ mice (Fig. 1h and Supplementary Fig. 1a–d). cGAS, STING and γH2AX levels were all increased in the hippocampi of 8-month-old ADLP^APP/PS1^ mice. However, in the brains of 4.5-month-old ADLP^APP/PS1^ mice, in which cGAS and STING protein levels were already increased, γH2AX levels did not differ from those in the brains of WT mice. Moreover, there were no significant changes in the γH2AX levels in the brains of 9-month-old App^NL-G-F^ mice. (Supplementary Fig. 1e–g). These results indicate that cGAS and STING activation in AD may not depend solely on the accumulation of nuclear DNA damage. Although often overlooked, immunostaining has shown that neuritic Aβ plaques frequently contain nucleic acids, as pointed out in a previous study^30^ (Supplementary Fig. 2a, b). In addition, Aβ can induce pathological neuritic plaque-dependent tau conversion^31^, and microglia-engulfed tau has been reported to induce PQBP1-dependent cGAS activation^32^. Immunostaining of the brains of dKI mice confirmed the presence of dsDNA and p-tau within the Aβ plaques (Fig. 1i). These results suggest a possible cell-extrinsic mechanism of cGAS activation in plaque-clustered microglia.

### Aβ and tau increased cytoplasmic DNA levels and induced distinct activation of microglia, which is dependent on STING

**Fig. 2:**
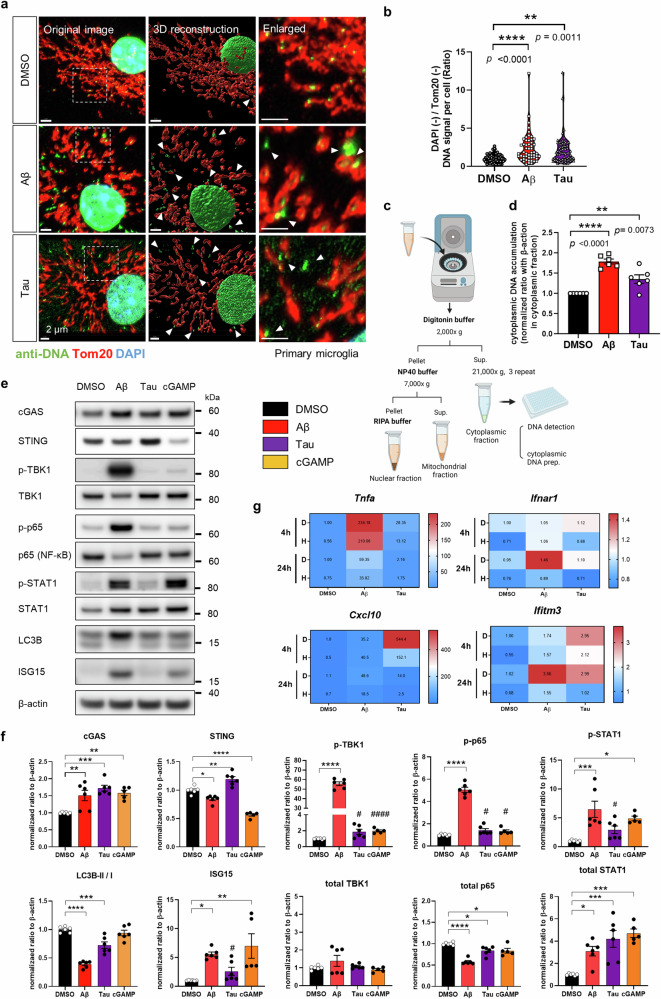
Aβ and tau both induced ectopic DNA accumulation and cGAS-STING pathway activation through different mechanisms in microglia. **a** Representative immunostaining images of DNA and Tom20 in primary microglia treated with Aβ and tau for 24 h. The arrow indicates ectopic DNA in the cytoplasm. Scale bars, 2 μm. **b** Quantification of DAPI- and Tom20-negative DNA signals in the cytoplasm of microglia. *n* = 51, 53, 72, *N* = 5 for each group. **c** Scheme of the subcellular fractionation of primary microglia. **d** Quantification of cytoplasmic dsDNA accumulation in Aβ- and tau-treated primary microglia. The cytoplasmic dsDNA signal was normalized to the β-actin immunoblot signal from the cytoplasmic fraction. *N* = 6 for each group. **e** Representative immunoblots of cGAS-STING pathway proteins in primary microglia treated with Aβ, tau, and cGAMP for 24 h. **f** Quantification of cGAS, STING and downstream molecules. *N* = 6,6,6,5. **g** Heatmap of the expression patterns of proinflammatory cytokines and type-I-interferon response genes in Aβ- and tau-treated primary microglia. H: H-151. n = 4 for each group. In (**b**), (**d**), and (**f**), the data are presented as the mean ± SEM. Statistical significance was determined by Student’s *t-*test; #*p* < 0.05. ####*p* < 0.0001., and one-way ANOVA with Tukey’s multiple comparisons test. *p < 0.05. **p < 0.01. ***p < 0.001. ****p < 0.0001. n indicates individual cells, *N* indicates individual biological replicates.

In nature, cytoplasmic DNA originating from the nucleus or mitochondria can activate cGAS^13^. In microglia, both Aβ and tau induce mtDNA release into the cytoplasm. In particular, donut- and oval-shaped mitochondria were mainly observed after Aβ treatment. (Fig. 2a). Tom20- and DAPI-negative DNA signals in the cytoplasm were significantly increased in both Aβ- and tau-stimulated microglia (Fig. 2d). In addition, dsDNA in the cytoplasmic fraction was measured with the ultrasensitive dsDNA dye PicoGreenI. Compared with control microglia, Aβ- and tau-treated microglia contained significantly more cytoplasmic DNA (Fig. 2e, f). Thus, both Aβ and tau were able to increase ectopic DNA in the cytoplasm, while Aβ induced clear morphological changes in mitochondria.

Next, we investigated cGAS-STING pathway molecules in microglia using cGAMP as a positive control. Aβ and tau induced different profiles of GAS-STING downstream activation. cGAS was upregulated by both Aβ and tau, but STING was slightly downregulated by Aβ but upregulated by tau. TBK1 and p65 phosphorylation were strongly activated by Aβ, but their phosphorylation was mild in tau-treated microglia. Both Aβ and tau induced IRF3 and STAT1 phosphorylation (Fig. 2e, f). In terms of gene expression profiles, Aβ induced a proinflammatory response followed by a type-I-IFN response, while tau induced a type-I IFN-skewed response. The time course of the responses was measured by *Tnfa*, *Cxcl10, Ifnar1*, and *Ifitm3* expression at the 4- and 24 h timepoints. STING inhibition had a negligible effect on Aβ-induced gene expression at the early timepoint but effectively attenuated persistent proinflammatory and type I IFN responses at the 24 h timepoint. Tau-induced gene expression was effectively reduced at both time points (Fig. 2g). These data indicate different responses of microglial STING to Aβ and tau.

**Fig. 3:**
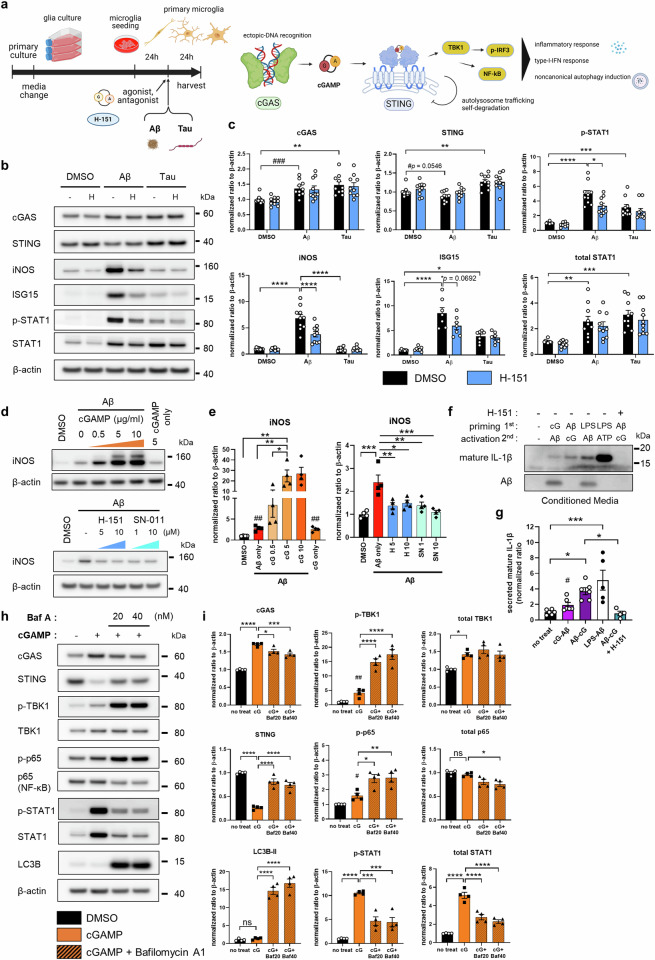
The neuroinflammatory microglial response is dependent on STING activity. **a** Schematic diagram of mouse primary microglial treatment and cGAS-STING pathway effector responses. **b** Representative immunoblots showing the phosphorylation of cGAS, STING, iNOS, ISG15, and STAT1 in Aβ- and tau-treated microglia. H: H-151. **c** Quantification of total protein and phosphorylation levels. *N* = 7 for ISG15, *N* = 10 for the other proteins. **d** Representative immunoblots showing changes in iNOS levels in Aβ-exposed microglia pretreated with a STING agonist or antagonist. **e** Quantification of iNOS protein levels. cG: cGAMP, H: H-151, SN: SN-011. *N* = 4 for each group. **f** Representative mature IL-1β blot indicating NLRP3 activation. **g** Quantification of mature IL-1β in microglia-conditioned media by blot analysis. *N* = 6,6,6,5,4. **h** Representative immunoblots of cGAS-STING pathway proteins in primary microglia cotreated with cGAMP and bafilomycin A1 (BafA). **i** Quantification of protein levels in Fig. 3h. Baf: bafilomycin A1. *N* = 4 for each group. In (**c**), (**e**), (**g**), and (**i**), the data are presented as the mean ± SEM. Statistical significance was determined by Student’s *t-*test, #*p* < 0.05. ##*p* < 0.01. ###*p* < 0.001, one-way or two-way ANOVA with Tukey’s multiple comparisons test. *p < 0.05. **p < 0.01. ***p < 0.001. ****p < 0.0001. *N* indicates individual biological replicates.

We further investigated the impact of STING regulation (Fig. 3a). Among the downstream molecules of STING, the Aβ-induced proteins p-STAT1, inducible nitric oxide synthase (iNOS) and interferon stimulatory gene-15 (ISG15) were downregulated in H-151-treated microglia (Fig. 3b, c). TBK1, IRF3, and p65 phosphorylation showed relatively little change. (Supplementary Fig. 3a, b). Aβ-induced iNOS was upregulated by cGAMP in a dose-dependent manner, and the STING antagonists H-151 and SN-011 both robustly decreased iNOS levels (Fig. 3d, e). We next investigated the NLRP3 inflammasome in microglia because STING has been reported to drive NLRP3 activation in peritoneal macrophages^33^^,^ and NLRP3 is critical for AD pathogenesis^34^. Intriguingly, sequential Aβ and cGAMP stimulation was sufficient to cleave mature IL-1β, a marker of NLRP3 activation. These data indicate that Aβ and cGAMP could function as physiological NLRP3 priming/activation cues in the context of AD. H-151 treatment completely blocked this process (Fig. 3f, g). In summary, NLRP3 activation and proinflammatory and interferon responses are mitigated by targeting STING. In addition, despite Aβ-induced cGAS-STING activation, incomplete degradation of STING has raised the question of how STING degradation is disrupted compared to the clear STING depletion by cGAMP. Aβ aggregates can overload or even inhibit lysosomal degradation, which is required for STING degradation^35^. Microglia treated with a combination of cGAMP and the autolysosome inhibitor bafilomycin A1 displayed STING and downstream kinase profiles very similar to those of Aβ-exposed microglia (Fig. 3h, i).

### STING knockout reversed the reduced reactivity of *APOE* ε4 human iPSC-derived microglia

Several genetic risk factors for AD are also involved in impaired endolysosomal flux. In particular, the *APOE4* genotype has been reported to impair cholesterol trafficking and activate interferon pathways in human microglia^36^. We investigated the impact of the *APOE* ε4 allele, the single strongest genetic risk factor for late-onset AD (LOAD), to determine whether the cGAS-STING pathway is also involved in LOAD. Human iPSC-derived microglia-like cells (iMGs) with *APOE* ε3 and ε4 alleles and an isogenic background were generated as described in a previous study^27^. Surprisingly, the levels of the cGAS and STING proteins were almost twice as high in *APOE* ε4/ε4 iMGs than in ε3/ε3 iMGs in the absence of any stimulation (Supplementary Fig. 4a, b). To further test the role of STING in ε4 iMGs, genetic ablation of STING in human ε4 iPSCs was achieved using CRISPR. Interestingly, both iPSCs and iMGs expressed cGAS, but differences in cGAS expression were detected only in iMGs. STING expression was limited in iMGs (Supplementary Fig. 4c, d). In the basal state, ε4 iMGs showed higher *IFNB1* gene expression than ε3 iMGs. When iMGs were stimulated with Aβ, ε4 iMGs expressed *TNFA* than ε3 iMGs. STING-KO ε4 iMGs tended to abrogate this phenotype (Supplementary Fig. 4e). These results indicate that the cGAS-STING pathway may be involved in reduced ε4 microglial responsiveness and may be affected by the *APOE* ε4 allele in addition to Aβ, tau, and age.

### Neuronal STING is upregulated in the brains of AD model mice

**Fig. 4:**
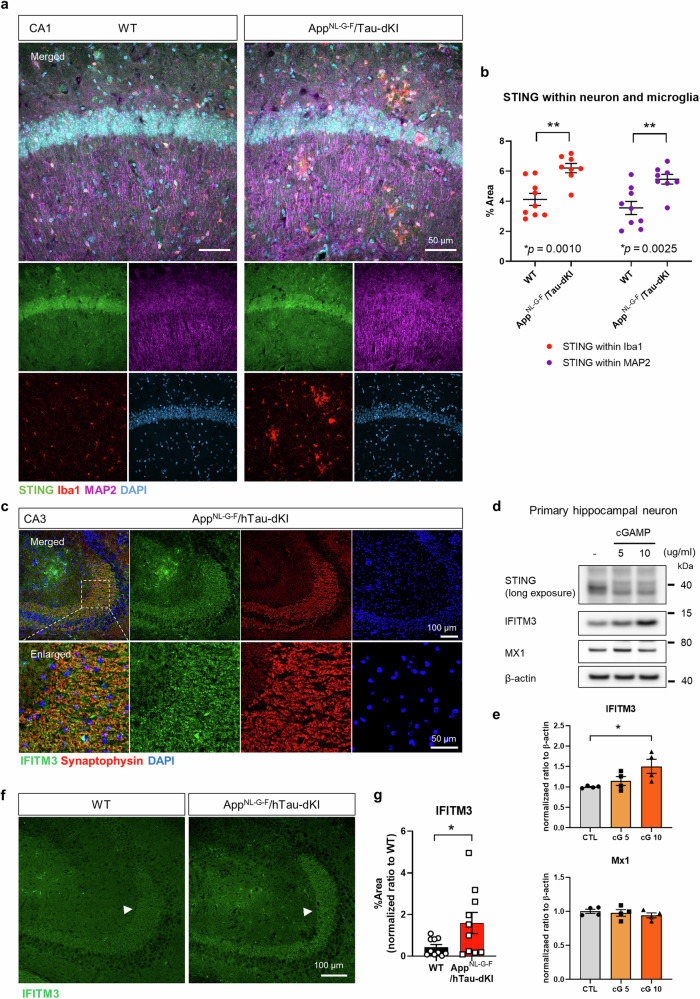
Neuronal STING is upregulated in the brains of AD model mice, and cGAMP can upregulate neuronal IFITM3. **a** Representative images of STING, Iba1, and MAP2 immunostaining in the hippocampal CA1 region of App^NL-G-F^/hTau-dKI mice. Scale bars, 50 μm. **b** Quantification of STING fluorescence intensity within MAP2- and Iba1-positive regions. **c** Representative immunostaining image of IFITM3 and the presynaptic marker Synaptophysin in the dKI brain CA3 region. **d** Representative immunoblots of the interferon-stimulatory proteins IFITM3 and MX1 in primary neurons after 24 h of cGAMP stimulation. **e** Quantification of neuronal IFITM3 and MX1 induction by cGAMP. *N* = 4 for each group. **f** Representative IFITM3 immunostaining in the brains of 12-month-old WT and dKI mice. The arrow indicates the CA3 mossy fiber neuronal region. Scale bars, 100 μm, 50 μm. **g** Quantification of the IFITM3 staining signal. *N* = 10,10.(**b**), (**e**), (**g**) Data are presented as the mean ± SEM. Statistical significance was determined by Student’s *t-*test and one-way ANOVA with Tukey’s multiple comparisons test. *p < 0.05. **p < 0.01. *N* indicates individual mice for (**b**) and (**g**) and biological replicates for (**e**).

cGAS and STING were most highly expressed in primary microglia, followed by astrocytes and neurons (data not shown). Surprisingly, however, significant STING expression was observed in neurons but not in astrocytes in vivo (Fig. 1c, Fig. 4a). STING was significantly upregulated in both neurons and microglia in the brains of App^NL-G-F^/hTau-dKI mice (Fig. 4b). Similar results were observed in the brains of App^NL-G-F^ mice and ADLP^APP/PS1^ mice (Supplementary Fig. 5a–c). The interferon-stimulated gene product IFITM3 was expressed in glia and neurons, especially in CA3 mossy fibers, in vivo (Fig. 4c). One study reported that neuronal IFITM3 can increase Aβ production under inflammatory conditions by modulating γ-secretase^37^. To determine whether neuronal IFITM3 could be induced by STING activation, we treated primary neurons with cGAMP. Although they had very low amounts of STING, neurons showed an increase in IFITM3 in response to cGAMP, whereas another interferon-inducible protein, MX1, was not affected (Fig. 4d, e). In addition, dKI mice displayed stronger IFITM3 signals than WT mice (Fig. 4f, g). To summarize, neuronal STING is upregulated in the brains of AD model mice, and STING activation can induce IFITM3 expression.

### Pharmacological STING inhibition mitigated brain inflammation with unexpected microglial transcriptomic changes

**Fig. 5:**
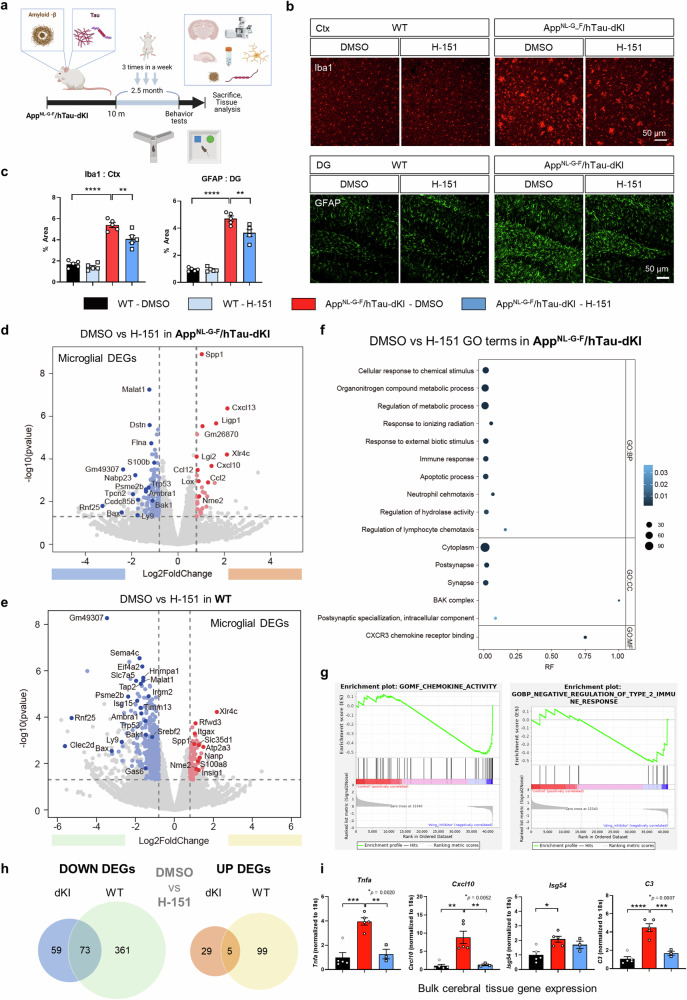
STING inhibition mitigated brain inflammation in App^NL-G-F^/hTau-dKI mice. **a** Scheme for the study design of pharmacological STING inhibition in dKI mice. **b** Representative Iba1 and GFAP immunostaining images showing alterations in microgliosis and astrogliosis. Scale bars, 50 μm. **c** Quantification of the Iba1+ and GFAP+ areas in the brains of dKI mice. *n* = 5 for each group. **d** Volcano plot of DEGs between brain-isolated microglia from DMSO- and H-151-injected dKI mice. *N* = 3,3. **e** DEGs between microglia from DMSO- and H-151-injected WT mice. Pastel red and blue dots represent genes with a log2FC > |0.8| and a *p-*value < 0.05, respectively. Selected genes are further highlighted, and other genes are presented as gray dots. *N* = 3,3. **f** Highlighted Gene Ontology (GO) terms of 166 STING-dependent DEGs in dKI mice. **g** Representative GSEA enrichment plots of STING-dependent DEGs in dKI mice. **h** Venn diagram of the shared upregulated and downregulated microglial DEGs after STING inhibition. **i** Cerebral brain bulk tissue gene expression in DMSO- and H-151-treated WT and dKI mice. *N* = 5,5,3.In **c** statistical significance was determined by two-way ANOVA with Tukey’s multiple comparisons test. **p < 0.01. ****p < 0.0001. In (**i**), statistical significance was determined by one-way ANOVA with Tukey’s multiple comparisons test. *p < 0.05. **p < 0.01. ***p < 0.001. ****p < 0.0001. *N* indicates individual mice.

To confirm whether the inhibition of STING could ameliorate AD pathology in vivo, we administered the STING inhibitor H-151 to 10-month-old dKI mice. We focused on KI models to exclude the potential influence of genetic defects in autophagy caused by *PSEN1* mutation on STING homeostasis. The permeability of the blood‒brain barrier (BBB) to cGAS-STING agonists and antagonists was simulated with an AlzPlatform BBB predictor (Supplementary Fig. 6a)^38^. H-151 was selected due to its BBB penetrability and applicability to both mice and humans^39^. H-151-treated dKI mice were further analyzed after behavioral tests (Fig. 5a). The reduction in brain p-TBK1 levels confirmed that H-151 was able to cross the BBB and affect the brain (Supplementary Fig. 6b, c). No significant weight loss was observed after injection (Supplementary Fig. 7a). We first examined glial reactivity. Microgliosis and astrogliosis were significantly ameliorated in the hippocampus and cortical areas after STING inhibition (Fig. 5b, c and Supplementary Fig. 7b). Then, we investigated the transcriptomic changes in microglia isolated from the brain. Since the identification of disease-associated microglia^40,41^, numerous attempts have been made to identify diverse microglial states and key drivers in AD and aging^42,43^. Although not popular, the interferon-responsive microglial state has been steadily reported, and this state is not an artifact of ex vivo activation^25^. Since the cGAS-STING pathway is required for the dsDNA-induced type-I-IFN response and recent cGAS manipulation effectively abrogated the microglial IFN signature^20^, we expected that STING inhibition would reduce the microglial IFN response. Potential ex vivo activation was inhibited as described in a previous study^25^ (Supplementary Fig. 7c).

We identified 34 upregulated and 132 downregulated genes between DMSO- and H-151-injected dKI mice. In the WT mouse group, 434 genes were upregulated (Supplementary Fig. 7d, Supplementary Table 2). We used *P* < 0.05 and |log2FC |>0.8 as cutoffs for differentially expressed genes (DEGs). Unexpectedly, some interferon-stimulating (*Oasl2, Ligp1*) and chemokine (*Cxcl13, Cxcl10, Ccl2, Ccl12*) genes were highly expressed in the H-151-injected dKI group. In the WT mouse group, *Xlr4c, Atp2a3, Rfwd3*, and *Insig1* were upregulated, while *Malat1, Rnf25, Bax, Ly9*, and *Isg15* were downregulated (Fig. 5d, e). To understand which biological pathways are affected by STING inhibition under AD conditions, we performed a GO enrichment analysis of the DEGs between the dKI-DMSO group and the H-151 group. GO analysis revealed STING-dependent DEGs related to the BAK complex, CXCR3 chemokine receptor binding, lymphocyte chemotaxis regulation, synapses, and postsynapse-related pathways (Fig. 5f). In line with the GO analysis results, the GSEA results showed that chemokine activity, the type 2 immune response, and the response to interferon-beta are regulated in microglia by STING inhibition (Fig. 5g, Supplementary Fig. 7e). DEGs shared by both the WT and dKI groups were considered common STING-dependent microglial transcripts. Five transcripts, *Spp1*, *Xlr4c*, *S100a8*, *Nme2*, and *Zfp984*, were shared upregulated DEGs, and 73 transcripts, including *Malat1*, *Rnf25*, *Gm49307*, *Bax*, *Bak1*, *Romo1*, *Lamtor2*, and *Hdac8*, were shared downregulated DEGs (Fig. 5h, Supplementary Fig. 7f). GO analysis highlighted B-cell negative selection, the BAK complex, the T-cell apoptotic process, mitochondrial membrane organization, and neurodegenerative disease-related pathways among the shared DEGs (Supplementary Fig. 7g). *Cxcl10*, *Ccl12*, *Iigp1*, *Lox2*, and *Spp1* were highly expressed in AD microglia and further upregulated after H-151 administration (Supplementary Fig. 7h). This result seemed contradictory because CXCL10 is a typical downstream cytokine of STING, and STING inhibition reduced its gene expression in primary microglia (Fig. 2g). However, although microglia express *Cxcl10*, brain staining revealed that the CXCL10 signal strongly overlapped with that of astrocytes and was reduced by H-151 (Supplementary Fig. 8a–d). When we measured whole cerebral tissue gene expression, *Tnfa*, complement component *C3*, and *Cxcl10* expression was significantly reduced after H-151 injection (Fig. 5i). Therefore, although it is difficult to determine why the levels of *Cxcl10* and a few other interferon-stimulated genes were elevated in microglia, the net effect of STING inhibition was to resolve brain inflammation. To summarize, peritoneal injection of the STING inhibitor H-151 induced an unexpected shift in the brain microglial transcriptome while reducing overall glial reactivity and brain inflammation. We also identified 78 STING-dependent microglial genes, including *Xlr4c* and *Gm49307*, whose biological functions are still undefined.

### STING inhibition ameliorated AD pathology and cognitive impairment in App^NL-G-F^/hTau-dKI mice

**Fig. 6:**
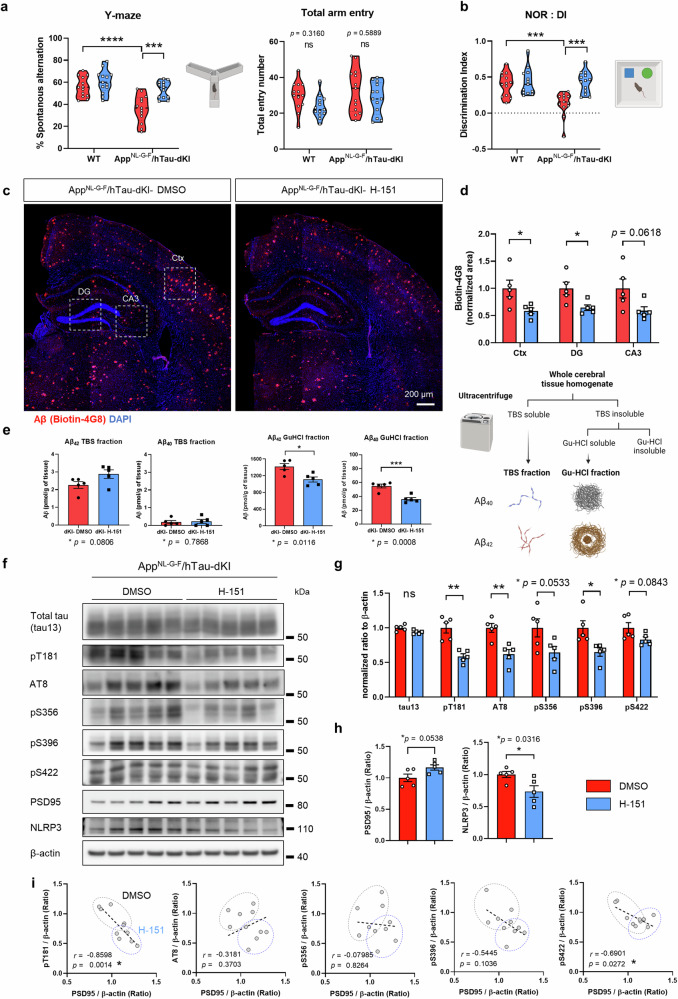
Pharmacological STING inhibition protected cognitive function and reduced Aβ and tau pathologies in App^NL-G-F^/hTau-dKI mice. **a** Percentages of spontaneous alternations and numbers of arm entries of DMSO-, H-151-injected WT and dKI mice in the Y-maze test. *n* = 13 for each group. **b** Novel object recognition test results for DMSO- and H-151-injected littermates and dKI mice. *n* = 13 for each group. **c** Representative immunostaining images of Aβ in the brains of DMSO- or H-151-treated dKI mice. The dashed line indicates the subregion used for quantification. Scale bar, 200 μm. **d** Quantification of Aβ-positive area proportions in brain subregions of DMSO-treated and H-151-treated dKI mice. *N* = 5,5. **e** The Aβ fractionation and ELISA quantification results for TBS-soluble, GuHCl-soluble Aβ_40_, and Aβ_42_ levels in whole cerebral brain tissue. *N* = 5,5. **f** Representative immunoblots of total tau, p-tau, PSD95, and NLRP3 proteins in cerebral brain tissue from DMSO- and H-151-treated dKI mice. **g** Quantification of total human tau (tau13) and tau phosphorylation at the pT181, AT8, pS356, pS396, and pS422 epitopes. *n* = 5 for each group. **h** Quantification of PSD95 and NLRP3 expression. *N* = 5,5. **i** Scatter plots for p-tau epitopes and PSD95 correlation. *N* = 5,5. In (**a**) and (**b**), the data are presented as the mean ± SEM. Statistical significance was determined by two-way ANOVA. ***p < 0.001. ****p < 0.0001. In (**d**), (**e**), (**g**), and (**h**), the data are presented as the mean ± SEM. Statistical significance was determined by two-tailed Student’s *t-*test. *p < 0.05. **p < 0.01. ***p < 0.001. In (**i**), the data are presented as the normalized ratio. Pearson’s correlation coefficient (*r)* was used to present correlation strength, and statistical significance was determined by two-tailed Student’s *t-*test. *p < 0.05. *N* indicates an individual mouse.

A pronounced decrease in memory and accumulation of Aβ plaques and hyperphosphorylated tau are key features of AD. We tested spatial working memory and object recognition memory function using the Y-maze and novel object recognition (NOR) behavioral tests. While control dKI mice exhibited memory impairment, H-151-injected dKI mice exhibited improved memory function (Fig. 6a, b). Aβ plaque coverage was significantly reduced in brain regions after STING inhibition (Fig. 6c, d). When we performed Aβ fractionation, the levels of soluble Gu-HCl and highly aggregated Aβ_40_ and Aβ_42_ were significantly reduced in whole cerebral brain tissue. The level of TBS-soluble Aβ_42_ tended to increase, but the difference was not significant (Fig. 6e). With respect to tau pathology, STING inhibition did not change the total tau level but significantly reduced tau phosphorylation. Moreover, hyperphosphorylated tau, rather than normal tau, contributes to synaptic dysfunction and loss. H-151-injected dKI mice had less p-tau at multiple epitopes, namely, pT181, AT8, pS356, pS396, and pS422 (Fig. 6f, g). Higher levels of the postsynaptic component PSD95 and had lower NLRP3 levels were observed in the in brains of STING-inhibited dKI mice (Fig. 6h). When we plotted a scatterplot to confirm the correlations, brain pT181 showed the strongest inverse correlation with PSD95. (Fig. 6i). The brain STING protein level was inversely correlated with the PSD95 level but positively correlated with the NLRP3 level (Supplementary Fig. 9a–c). To summarize, pharmacological inhibition of STING in dKI mice significantly reduced the Aβ burden and tau hyperphosphorylation and preserved memory function.

### STING inhibition reduced microglial p-STAT1 levels and synaptic engulfment in App^NL-G-F^/hTau-dKI mice

**Fig. 7:**
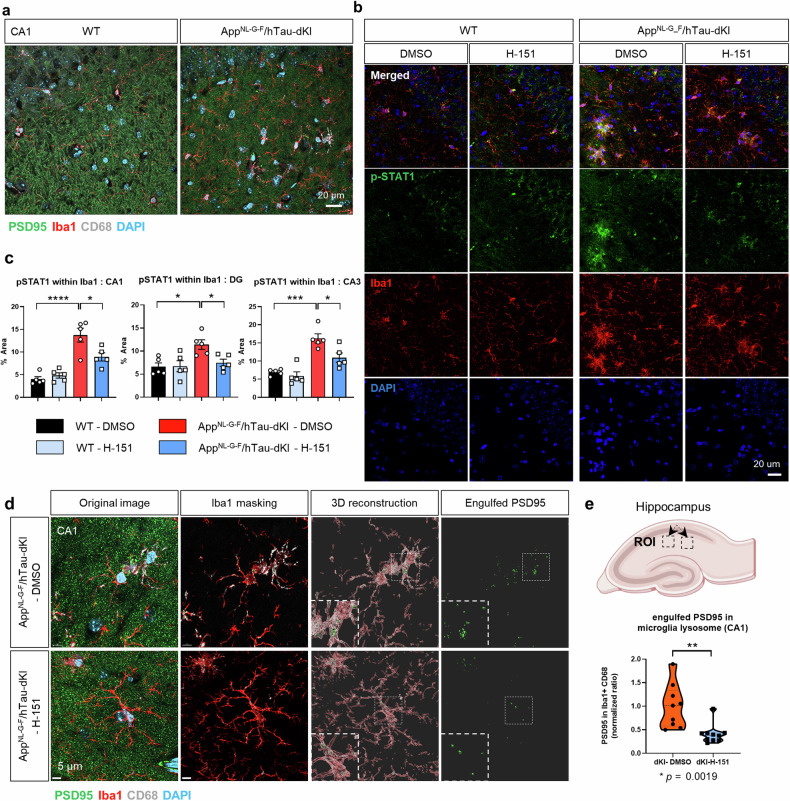
Pharmacological STING inhibition reduced microglial p-STAT1 levels and synapse engulfment. **a** Representative images of PSD95, CD68 and Iba1 immunostaining in the hippocampal regions of WT and dKI mice. Scale bars, 20 μm. **b** Representative images of p-STAT1 and Iba1 immunostaining for microglial p-STAT1. **c** Quantification of the p-STAT1 signal area within Iba1. *N* = 5 for each group. **d** Representative immunostaining and 3D-reconstructed images of PSD95, CD68, and Iba1 in the hippocampal CA1 region. Scale bars, 5 μm. **e** Region of interest and quantification results of microglia-engulfed PSD-95 volumes in the hippocampal CA1 region. *n* = 9, 10, *N* = 5,5.All the data are presented as mean ± SEM. In (**c**), statistical significance was determined by two-way ANOVA. *p < 0.05, ***p < 0.001. ****p < 0.0001. In (**e**), statistical significance was determined by two-tailed Student’s *t* test. **p < 0.01. n indicates biological replicates. *N* indicates an individual mouse.

In AD, excessive synapse loss is primarily caused by microglia and the complement system^44^. Recent studies have revealed the role of type I IFN signaling and p-STAT1-positive microglia in postsynaptic loss in a 5xFAD model^30,45^. Since the cGAS-STING pathway functions upstream of the type I IFN response, we hypothesized that postsynaptic microglial engulfment could be dependent on STING. Compared to WT mice with clear dendritic PSD95 patterns, dKI mice had disrupted PSD95 patterns and microglia with an enlarged CD68+ lysosomes (Fig. 7a). Microglial p-STAT1 levels were significantly reduced in the hippocampus by STING inhibition (Fig. 7b, c). We then examined postsynaptic microglial engulfment. Notably, H-151-treated dKI mice exhibited less microglial engulfment in the CA1 region than DMSO-injected mice (Fig. 7d, e). In summary, pharmacological inhibition of STING effectively reduced microglial p-STAT1 levels and postsynaptic engulfment, which is consistent with the results of the behavioral tests.

## Discussion

**Fig. 8:**
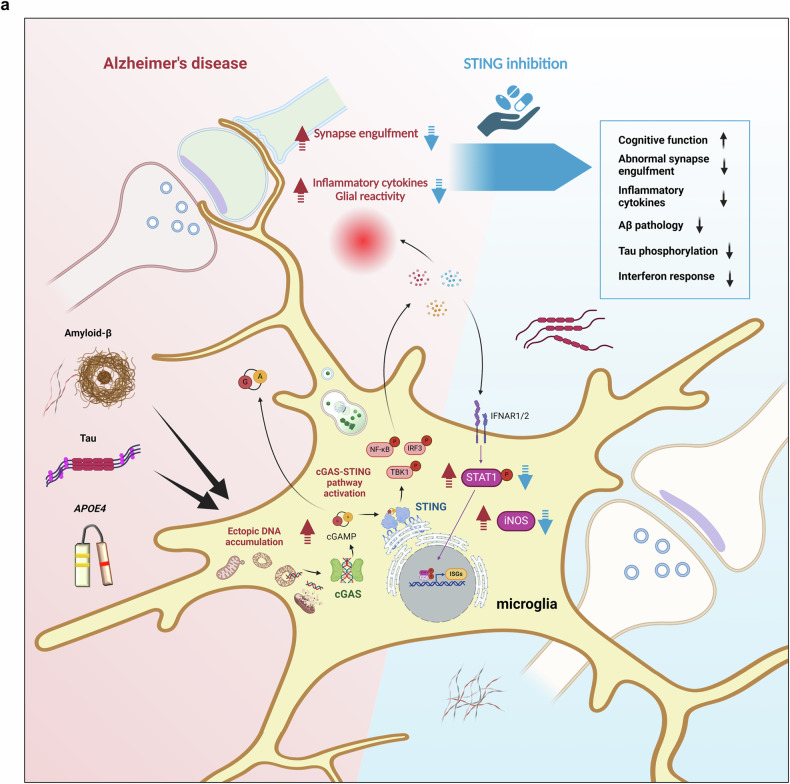
Graphical summary of the study. **a** Aβ, tau, and the *APOE* ε4 allele affect the cGAS-STING pathway in microglia. Aβ and tau increase cytoplasmic DNA accumulation and upregulate cGAS. cGAMP produced from cGAS diffuses and activates STING in surrounding brain tissue. Activated STING triggers an inflammatory response followed by an interferon response through downstream TBK1, NF-κB, IRF3, and p-STAT1 effectors. Pharmacological STING inhibition ameliorates a broad range of AD pathologies, including neuroinflammation, Aβ burden, phospho-tau levels, microglial synapse engulfment, and memory impairment.

Neurodegenerative disorders are associated with abnormal immunological function. We demonstrated the role of cGAS-STING innate immune pathway activation in AD, with an emphasis on STING. The cGAS-STING pathway was activated in AD brain tissue, and STING was required for Aβ and p-tau accumulation and microglial synapse engulfment (Fig. 8a). Both Aβ and tau, as well as the *APOE* ε4 allele, were linked to increases in cGAS and STING levels in human microglia. In vivo, neuronal STING was significantly upregulated, and STING activation in neurons increased IFITM3 expression. Two recent independent studies revealed the involvement of the cGAS-STING pathway in 5xFAD amyloidosis and *P301S* tauopathy mouse models, with a focus on cGAS manipulation^19,20^. Both amyloid and tau are key players in AD pathogenesis, and their interaction results in different outcomes than those induced by each factor individually. We showed that STING suppression was effective in AD model mice that expressed both Aβ and human tau. Furthermore, the relationship between microglial *APOE4* and the cGAS-STING pathway in AD has not been previously reported. Pharmacological targeting or genetic ablation of cGAS and STING tends to be protective in diverse neural disorders. However, it is still unknown which is more effective to target. Although targeting the upstream element cGAS may have broader protective effects than targeting STING, it also has the potential to unintentionally inhibit beneficial processes such as the DNA damage response.

Our data showed that STING inhibition reduced Aβ aggregation and tau phosphorylation (Fig. 6c–i). The decrease in p-tau levels after STING inhibition is noteworthy because Aβ-induced tau hyperphosphorylation is closely associated with synapse loss and memory impairment in AD, and pathological tau conformers are often localized in dendritic spines. Intriguingly, neurons containing pathological tau are exposed to phosphatidylserine and phagocytosed by microglia in an MFGE8- and NO-dependent manner^46^. Our data revealed that STING inhibition reduced iNOS induction (Fig. 3d, e) and inhibited microglial engulfment of postsynaptic material in the brains of dKI mice (Fig. 7d, e). Consistently, the STING inhibitor-treated mice exhibited preserved working memory and object recognition memory (Fig. 6a, b). Aβ-induced tau phosphorylation and conversion, dendritic tau accumulation, and abnormal microglial synapse engulfment are closely interrelated, and our data suggest that the cGAS-STING pathway is strongly involved in these phenomena.

We found that STING-dependent microglial transcripts and 78 DEGs were common to both WT and dKI mice (Fig. 5h). For example, among the shared downregulated DEGs, the lncRNA *Malat1* can regulate STING expression, and the E3 ubiquitin ligase *Rnf25* is involved in NF-κB signaling. Interestingly, *Bax* and *Bak1* have proapoptotic functions but can also form mitochondrial membrane pores for mtDNA efflux^47^. Whether STING downregulation can provide feedback to upstream processes remains an open question. Among the shared UP DEGs, the DAM-associated gene *Spp1*, encodes SPP1/osteopontin, an extracellular matrix protein involved in synapse remodeling^48^. One study revealed that *Spp1* can promote synapse loss in 6-month-old App^NL-F^ mice when its expression is restricted to perivascular macrophages and fibroblasts; however, the authors also noted its expression in the microglia of 15-month-old mice^48^. Additionally, because SPP1 has inflammation-modulating functions^49^, increased SPP1 expression can be beneficial in late-stage AD. However, functional studies of *Xlr4c* and *Gm49307* are lacking, and their relationship with STING has not been previously reported. *Xlr4c* has been reported to be transcriptionally repressed in the paternal allele^50^, making it potentially involved in sex-dependent microglial function.

Although microglial p-STAT1 levels were reduced after H-151 injection (Fig. 7b, c), transcriptomic analysis showed that considering p-STAT1 as a marker of the type-I-IFN response in microglia could be an oversimplified assumption. Previous single-cell analyses conducted in AD models have consistently shown that microglial subsets enriched in interferon-stimulated genes such as *Oasl1, Ifitm3, Isg15, Stat1*, and *Cxcl10* ^42,43^ are upregulated by cGAS-STING pathway activation in other diseases. Pharmacological STING inhibition unexpectedly increased the expression of several chemokines and interferon-stimulated genes in the microglia of dKI mice (Fig. 5d–g). In part because H-151 was administered intraperitoneally for 2.5 months, it has to be defined if chronic or peripheral effects may have contributed to this result.

The peripheral immune system is an underestimated factor of AD. However, recent studies have suggested that microglia-mediated T-cell infiltration contributes to neurodegeneration in AD^51,52^. Although the relationship between the cGAS-STING pathway and T-cell infiltration in the context of AD was not directly addressed in this study, our data, together with those of previous reports, may provide useful insights into this phenomenon. First, as we have shown, the cGAS-STING pathway is activated in the brains of multiple types of AD model mice, and chemokines such as CXCL10, which attracts infiltrating T cells, are known downstream effectors of STING. GO and GSEA results highlighted lymphocyte chemotaxis and CXCR3 chemokine receptor binding pathways (Fig. 5d–g). This result was based on the high levels of *Cxcl10*, *Cxcl13*, and *Ccl2* in microglia. However, the majority of CXCL10 was found in astrocytes (Supplementary Fig. 7a–d), and total cerebral *Cxcl10* expression was reduced by STING inhibition (Fig. 5i). In addition, endothelial STING has been reported to induce T-cell transendothelial migration via the CXCL10-CXCR3 axis^53^. Thus, theoretically, cGAS-STING pathway activation could be a potential cause of T-cell infiltration in the AD brain. Second, two studies have reported the protective and detrimental roles of brain-infiltrated T cells in different models of amyloidosis and tauopathy^51,52^. In our study, AD mice carrying both Aβ and tau were injected intraperitoneally with a STING inhibitor. Enriched GO terms of common DEGs included T-cell apoptotic process (Supplementary Fig. 7g), and T-cell-intrinsic STING function is required for prolonged function of memory CD8 + T cells^54^. Therefore, the protective effect of STING inhibition in our study could rely on peripheral regulation of STING activity. Further systematic analyses are need to confirm these results.

The cGAS-STING pathway connects immune activation to tissue deterioration. Aging is the most important risk factor for AD, and a recent notable study revealed that this pathway is crucial for age-induced neuroinflammation and neurodegeneration^28^. Destabilized heterochromatin and mtDNA accumulation increase with age. Both Aβ and tau increased the amount of cytoplasmic mtDNA (Fig. 2a–d) but through different kinetics and the activator of different downstream kinases(Fig. 2e–g). This result could be explained by the bypass of parallel signaling hubs. For example, Aβ can activate TLR4, which is located upstream of TBK1 and p65, causing excessive phosphorylation. Tau has been reported to activate cGAS via direct binding of PQBP1 or by causing mtDNA leakage^32^. TNF-α was strongly expressed early after Aβ exposure (Fig. 2g) and was reported to release mtDNA and activate cGAS^55^. Therefore, the status of cGAS-STING downstream kinases in Aβ-exposed microglia represents the combined impact of the activation of other proinflammatory pathways.

The maturation of IL-1β is dependent on NLRP3 activation, and STING activation leads to NLRP3 inflammasome activation. The utilization of LPS to induce inflammatory stimulation is part of NLRP3 priming/activation in vitro. However, it is difficult to believe that the normal brain contains LPS. Our earlier study demonstrated that LPS/Aβ can function as a prime/activation cue for NLRP3^56^ and one report suggested that cGAMP can function as both a prime and an activation cue for NLRP3^57^. Therefore, we hypothesized that combining cGAMP and Aβ would provide considerably more physiological cues for NLRP3 priming/activation. As a result, successive Aβ and cGAMP treatment induced IL-1β maturation and vice versa. The STING inhibitor H-151 completely inhibited this mechanism (Fig. 3f–g). The brains of App^NL-G-F^ mice contained significant levels of cGAMP and Aβ (Fig. 1g), while STING inhibition decreased brain NLRP3 protein levels (Fig. 6f, h). Thus, Aβ-GAMP may operate as a physiological priming and activation cue for NLRP3 in AD.

At least four cGAMP import routes have been identified thus far^58^. Notably, P2X7R, a previously reported potassium efflux channel for NLRP3 activation, has been identified as an ATP-assisted cGAMP-importing channel^79^. The intriguing nature of cGAS and STING in nonimmune cells is predicated on the paracrine of cGAMP. cGAMP produced from dying tumor cells, cGAS, is transferred to surrounding immune cells and leads to STING activation when efferocytosis is blocked in tumor-associated macrophages by an anti-MerTK antibody^59^. In the brain, Trem2, Dectin-1^60^, MerTK^61^ and other scavenging receptors have important functions in efferocytosis. One study indicated that degenerating neurons exhibit increased *Cgas* (*Mb21d1*) gene expression^62^. Although the nociceptive role of STING in peripheral neurons has been described previously^63^, the role of STING in brain neurons has rarely been addressed. Our data showed that STING was present at a significant level in neurons in vivo and that the level of neuronal STING was increased, similar to that of microglial STING, in the dKI brain (Fig. 4a, b). It is reliable to infer that activated STING in both neurons and glia may alter the AD brain environment. The cause of the increase in neuronal STING levels in the brain remains to be determined and is a limitation of this study.

Our data also showed that IFITM3 was upregulated by cGAMP in neurons (Fig. 4d, e). During viral infection, interferon-induced IFITM3 functions as a shuttle protein that sends viral cargo to lysosomes^64^. In AD, IFITM3 has been identified as a regulator of γ-secretase, which affects Aβ production in neurons^37^. Given that Aβ has an antimicrobial function and that the cGAS-STING pathway induces antiviral immune responses, it is possible that chronic cGAS-STING pathway activation may promote Aβ accumulation via IFITM3. Furthermore, the upregulation of cGAS and STING in basal-state *APOE* ε4 iMGs suggested that *APOE* ε4 carriers may be more sensitive to ectopic DNA (Supplementary Fig. 4a, b).

Despite the context of different diseases, such as AD, aging, cancer, and infection, our immune systems present similar biological mechanics and seem to maximize their efficiency by leveraging functional components that already exist. Here, we showed that cGAS-STING pathway activation in microglia and neurons is strongly associated with AD pathogenesis. First, Aβ, tau, and *APOE* ε4 allele increase the expression of cGAS and STING, and neuronal STING expression is increased in the AD brain. Second, STING was found to be strongly required for microglial proinflammatory activity, type-I-IFN responses and NLRP3 activation. IFITM3 expression was dependent on STING both in microglia and neurons. Finally, the Aβ burden, tau hyperphosphorylation and abnormal synapse loss, which are widespread pathological features of AD, were suppressed by pharmacological STING inhibition. Taken together, our findings shed light on the pathogenic mechanisms of STING in AD and the potential of STING as a therapeutic target.

## Supplementary information

Supplementary Information

Supplementary Table 1

Supplementary Table 2
