# Supplementary material for: Blockade of STING activation alleviates microglial dysfunction and a broad spectrum of Alzheimer’s disease pathologies
Source: Exp Mol Med. 2024 Sep 2;56(9):1936–51. doi: 10.1038/s12276-024-01295-y (PMC11447230; doi:10.1038/s12276-024-01295-y)
Supplement: Supplementary file 1 — Supplementary Information [file 12276_2024_1295_MOESM1_ESM.pdf]

Supplementary Information

Blockade of STING activation alleviates microglial dysfunction and a broad spectrum of Alzheimer's disease pathologies.

Sunwoo Chung[1,2], June-Hyun Jeong[1,2], Jong-Chan Park[3], Jong Won Han[1,2], Yeajina Lee[2,4], Jong-Il Kim[2,4], Inhee Mook-Jung[1,2]*

***Correspondence: Inhee Mook-Jung, Ph.D.**

Supplementary Materials and Methods

Supplementary Figures 1-9 with figure legends.

Author contributions

Supplementary Materials and Methods

Primary cell culture

Primary mouse microglia were obtained from P1 ICR mouse pup. Brains were dissected and meninges were fully removed in Ca^{2+}-, Mg^{2+}-free Hank's balanced salt solution (HBSS, Welgene) at 4°C. Chunks were transferred to Dulbecco's modified Eagle's medium (DMEM, Hyclone) supplemented with 10% fetal bovine serum (FBS, Gibco) and 1% penicillin and streptomycin (Sigma). Tissue was mechanistically dissociated by sequential pipetting using flame-polished Pasteur pipette. Cell suspension was than filtered through 40μm mesh filter and plated in poly-D-Lysine (PDL, Sigma)-coated T75 flask for mixed glia culture. Next day, culture medium was fully changed and Mixed culture was maintained until 10-11 days. Surface attached microglia were collected, and plated on PDL-coated culture plates and used within two days. Primary astrocytes were obtained from mixed glia culture before the induction of microglia. In day 7, culture medium was removed and cells were rinsed 2 times with DPBS then detached by 0.05% trypsin-EDTA solution. Reaction was stopped by adding FBS-supplemented DMEM. Collected cells were centrifuged, resuspended and plated in PDL-coated culture plate. In next day, culture medium was fully changed and astrocytes were used within 3 days. Primary neuron was cultured as in previous study [1]. Briefly, E16 ICR mouse pup hippocampi were dissected in Hibernate buffer. Cell suspension was obtained by papain digestion and mechanical dissociation. Suspension was filtered through 70μm mesh filter and plated in PDL-coated culture plates which filled with B27-supplemented neurobasal medium-A (Gibco). The half of medium was changed for every 3 days until the day 14. All cell culture was maintained in a humidified incubator at 37°C with 5% CO_2.

Amyloid-β (Aβ) peptide preparation

$A\beta_{1-42}$ synthetic peptide (Bachem, H-8146) was dissolved in 1,1,1,3,3,3-Hexafluoro-2-propanol (Merck) for 1 mg/ml concentration and incubated at room temperature for 72 hours on a rotating shaker. Incubated peptide solution was dispensed into tubes using gas tight syringe (ILS) and lyophilized. Lyophilized peptides were stored at -80°C until use. Aβ was dissolved in anhydrous dimethyl sulfoxide (DMSO) before use.

Brain tissue preparation

Mice were anesthetized and perfused with ice-cold PBS. brain parts were snap-frozen and stored at - 80°C before further analysis. Brain tissue samples were first homogenized in Tris buffer solution (TBS; 25mM Tris-HCl, 150mM NaCl, pH 7.4, 1mM EDTA, 1mM EGTA) supplemented with RNase inhibitor (Roche), phenylmethylsulfonyl fluoride (PMSF, Sigma), protease inhibitor cocktails (PI, Sigma) and phosphatase inhibitor cocktails-I, II (PPI-I, II, A.G. scientific) for simultaneous analysis. Homogenates were then immediately aliquoted for RNA extraction, protein sampling, and Aβ fractionation. For immunostaining, brain hemispheres were fixed in 4% PFA for 24 hours at 4°C and then in 30% sucrose (w/v) in PBS for 72 hours. Frozen brain tissues embedded in OCT compound were coronally sectioned at 30 μm thickness using a CM 1850 Cryostat (Leica), and stored in tissue storage buffer at 4°C until use.

Gene expression analysis

RNA was extracted by using a RNeasy PLUS Mini kit (QIAZEN). cDNA was synthesized from 150ng of total RNA using a Maxime RT PreMix kit, (iNtRON Biotechnology). Quantitative real-time PCR was performed by utilizing KAPA SYBR® FAST qPCR ABI Prism kit (KAPA

Biosystems). Expression levels of target mRNAs were analyzed by using the ΔΔCt method and normalized to the expression level of *Rps18*. Fold changes are displayed as a relative ratio to the control group.

Blood-Brain-Barrier (BBB) permeability prediction test

BBB permeability of agonists and antagonists were tested by AlzPlatform BBB predictor webtool (https://www.cbligand.org/BBB/index.php)[2]. Compound chemical structures from PubChem were used as an input. Support vector machine (SVM) algorithm was applied for the chemical property prediction.

Supplementary Figures

Supplementary Fig. 1

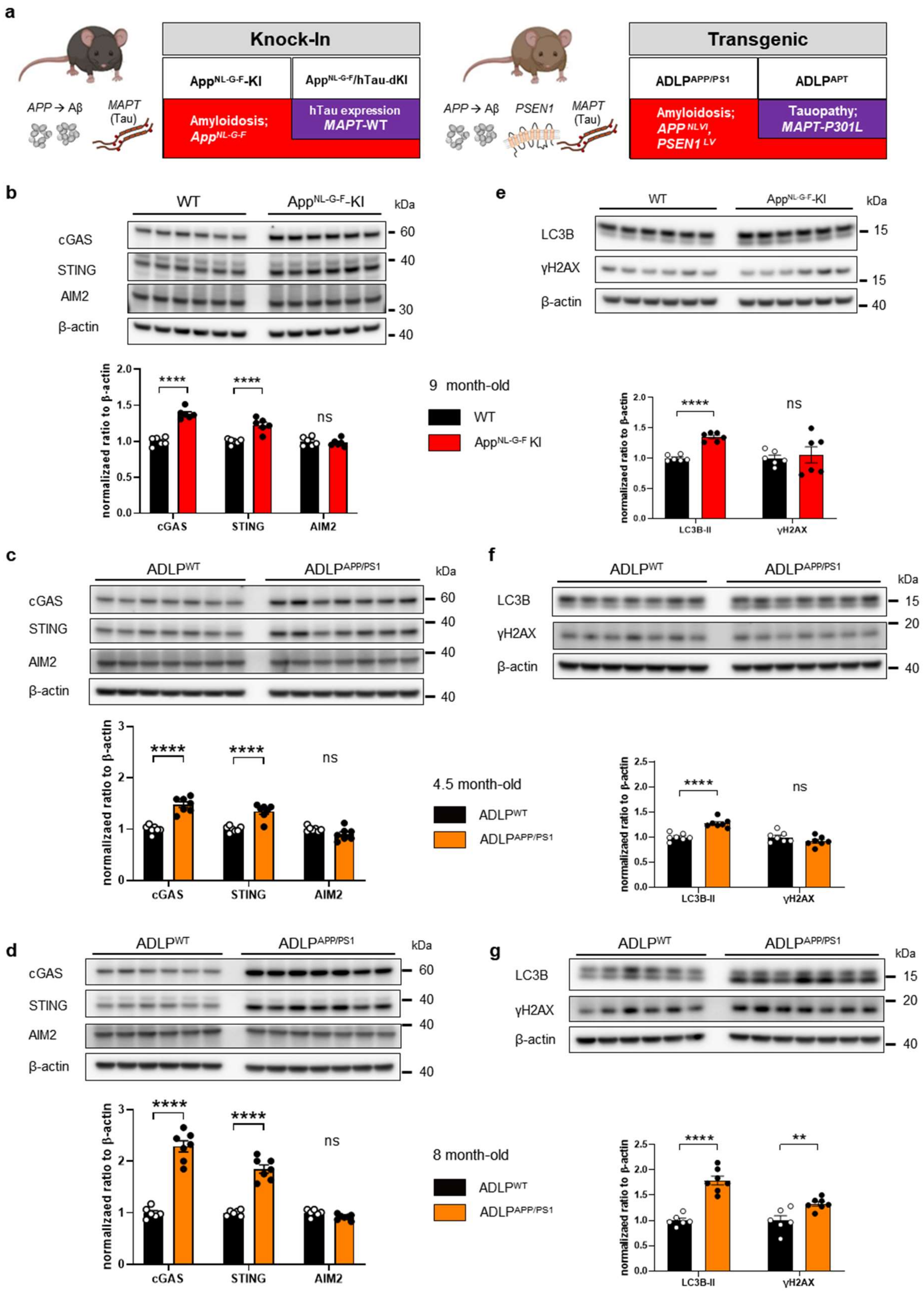

Supplementary Fig. 1. Time-course of cGAS, STING, and γH2AX brain accumulation in AD model mice.

a, Graphical summary of *App, MAPT* knock-in (KI) and ADLP transgenic AD model mice which used in the study. **b**, Representative immunoblots and quantification results of cGAS, STING, and AIM2 protein levels in 9-month-old App$^{NL\text{-}G\text{-}F}$ mice. *N*=6,6. **c**, Representative immunoblots and quantification results of cGAS, STING, and AIM2 protein levels in 4.5-month-old ADLP$^{APP/PS1}$ mice. *N*=7,7. **d,** Representative immunoblots and quantification results of cGAS, STING, and AIM2 protein levels in 8-month-old ADLP$^{APP/PS1}$ mice. *N*=6,7. **e,** Representative immunoblots and quantification results of LC3 and γH2AX protein levels in 9-month-old App$^{NL\text{-}G\text{-}F}$ KI mice. *N*=6,6. **f,** Representative immunoblots and quantification results of LC3 and γH2AX protein levels in 4.5-month-old ADLP$^{APP/PS1}$ mice. *N*=7,7. **g,** Representative immunoblots and quantification results of LC3 and γH2AX protein levels in 8-month-old ADLP$^{APP/PS1}$ mice. *N*=6,7.

All data are presented as mean ± s.e.m. Statistical significance was determined by two-tailed Student's t-test. $^{**}p < 0.01$, $^{****}p < 0.0001$.

Supplementary Fig. 2

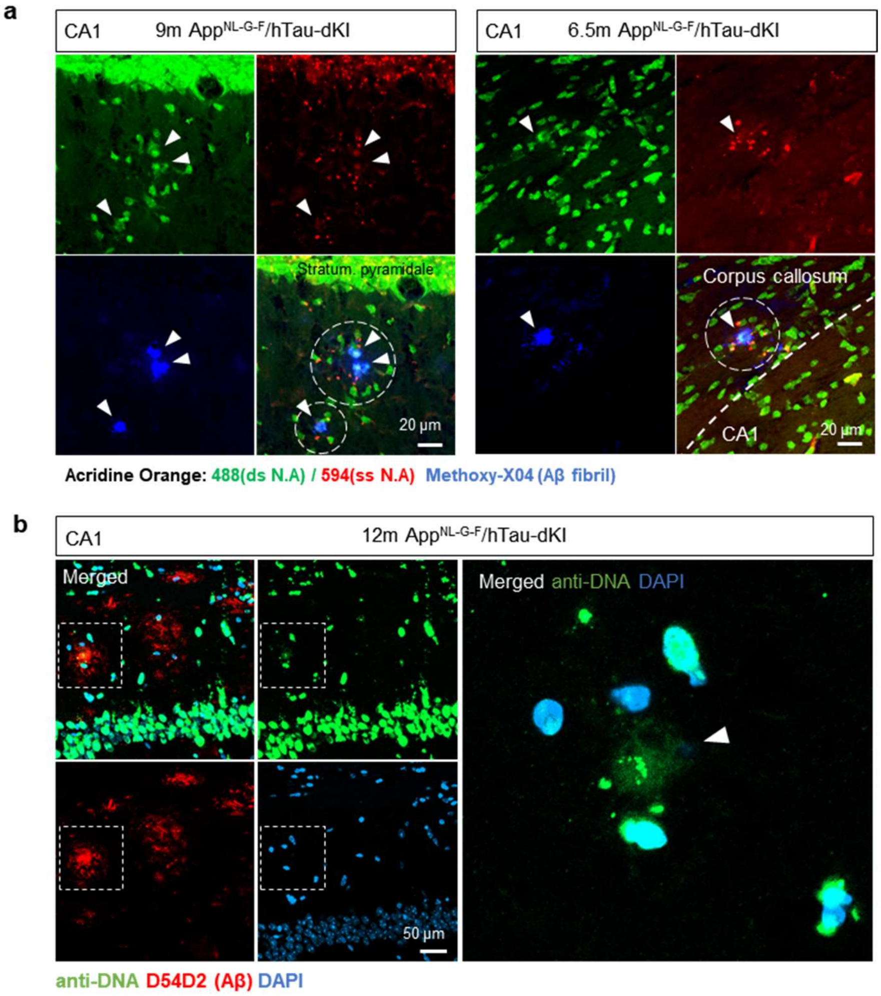

Supplementary Fig. 2. Nucleic-acid positive Aβ plaques in AD model mice.

a, Nucleic-acid dye acridine orange (AO) and fibrillar Aβ dye methoxy-X04 co-stained image in App$^{NL\text{-}G\text{-}F}$/hTau-dKI mice and App$^{NL\text{-}G\text{-}F}$ mice brain CA1 region. AO 488nm excitation signal indicates double-stranded nucleic acid and 594nm excitation signal indicates single-stranded nucleic acids. Scale bars, 20 µm. **b,** Immunostaining image for Aβ and DNA in App$^{NL\text{-}G\text{-}F}$/hTau-dKI mice. Arrowhead in enlarged image indicates DNA immune-stained signal in Aβ plaque which is negative for DAPI. Scale bars, 50 µm

Supplementary Fig. 3

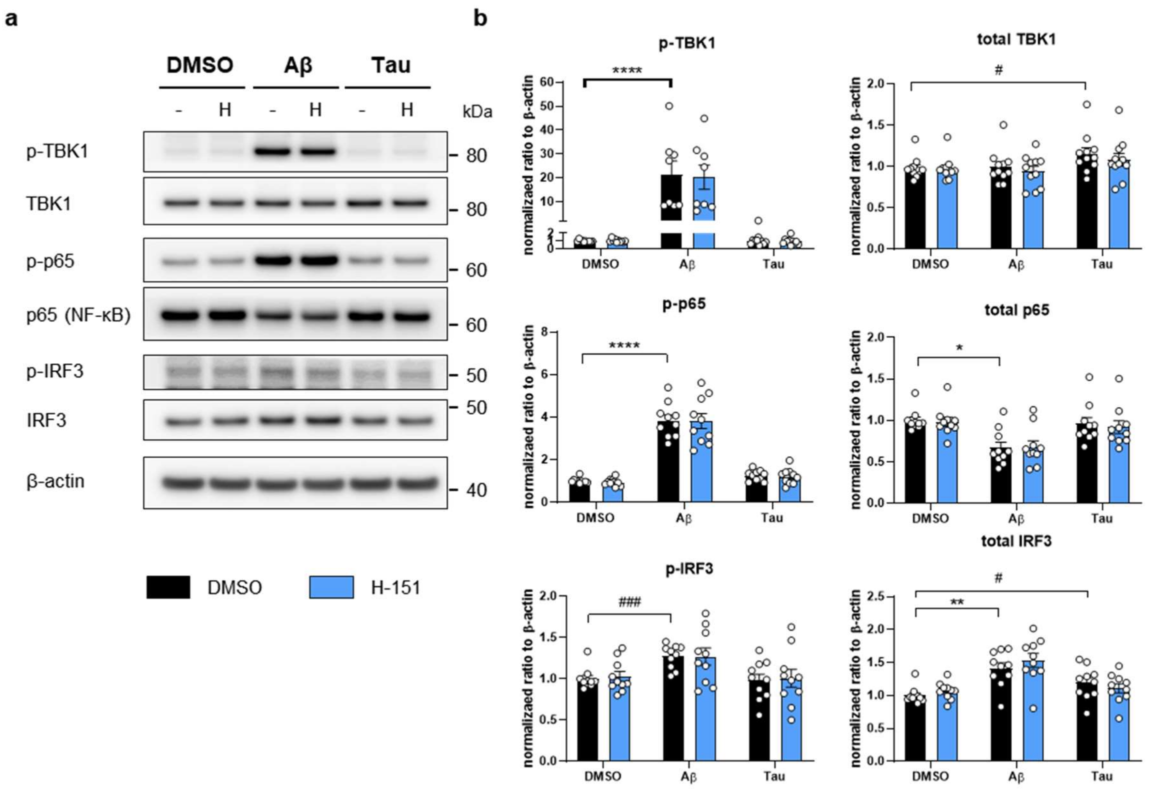

Supplementary Fig. 3. Aβ and tau induced cGAS-STING pathway signaling molecular patterns.

a, Representative immunoblots of cGAS-STING downstream molecules TBK1, p65 and IRF3 and their phosphorylation. Related with Fig. 3 b-c. **b,** Quantification results of TBK1, p65 and IRF3 and their phosphorylation levels. *N*=10, 10 for TBK1 and NF-κB, *N*=10 for each group.

In (**b**), data are presented as mean ± s.e.m. Statistical significance was determined by two-way ANOVA with Tukey's multiple comparisons test. *$p < 0.05$. **$p < 0.01$. ****$p < 0.0001$. and Student's t-test. #$p < 0.05$. ###$p < 0.001$. *N* indicates individual biological replicates.

Supplementary Fig. 4

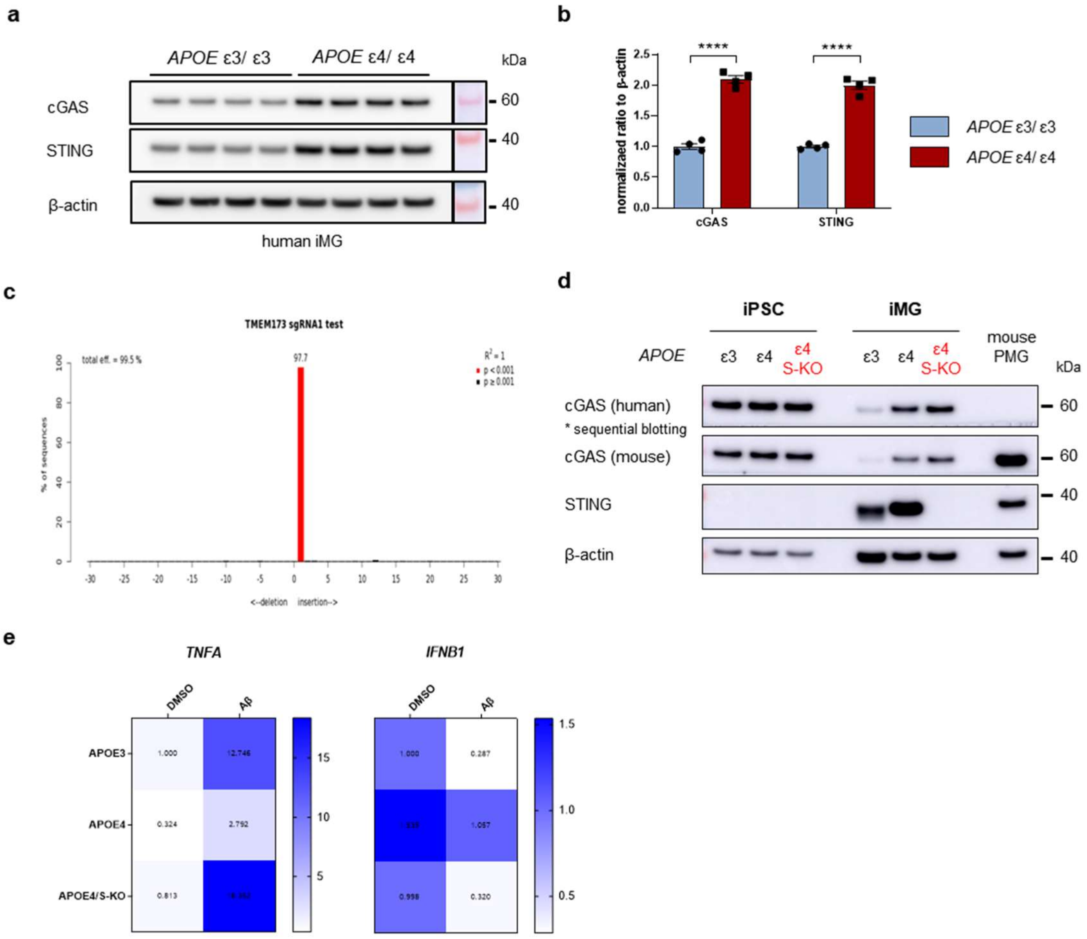

Supplementary Fig. 4. STING-knockout reverses blunted reactivity of *APOE* ε4 human iPSC-derived microglia.

a, Immunoblots of cGAS and STING in human iPSC-derived microglia-like cells (iMG) with ApoE ε3 and ε4 alleles in isogenic background. **b**, Quantification of cGAS and STING protein levels in iMG. N=4,4. **c**, Insertion/deletion analysis result of sgRNA targeted *TMEM173* sequence in STING-knockout (S-KO) iPSC. **d,** Representative immunoblots of cGAS, STING and confirmation of S-KO *APOE* ε4 iMG. **e**, *TNFA* and *IFNB1* gene expression levels in Aβ-stimulated *APOE* ε3, ε4, and ε4 STING-KO human iMG.

In (**b**), Data are presented as mean ± s.e.m. Statistical significance was determined by Student's t-test. ****p < 0.0001. N indicates individual biological replicates.

Supplementary Fig. 5

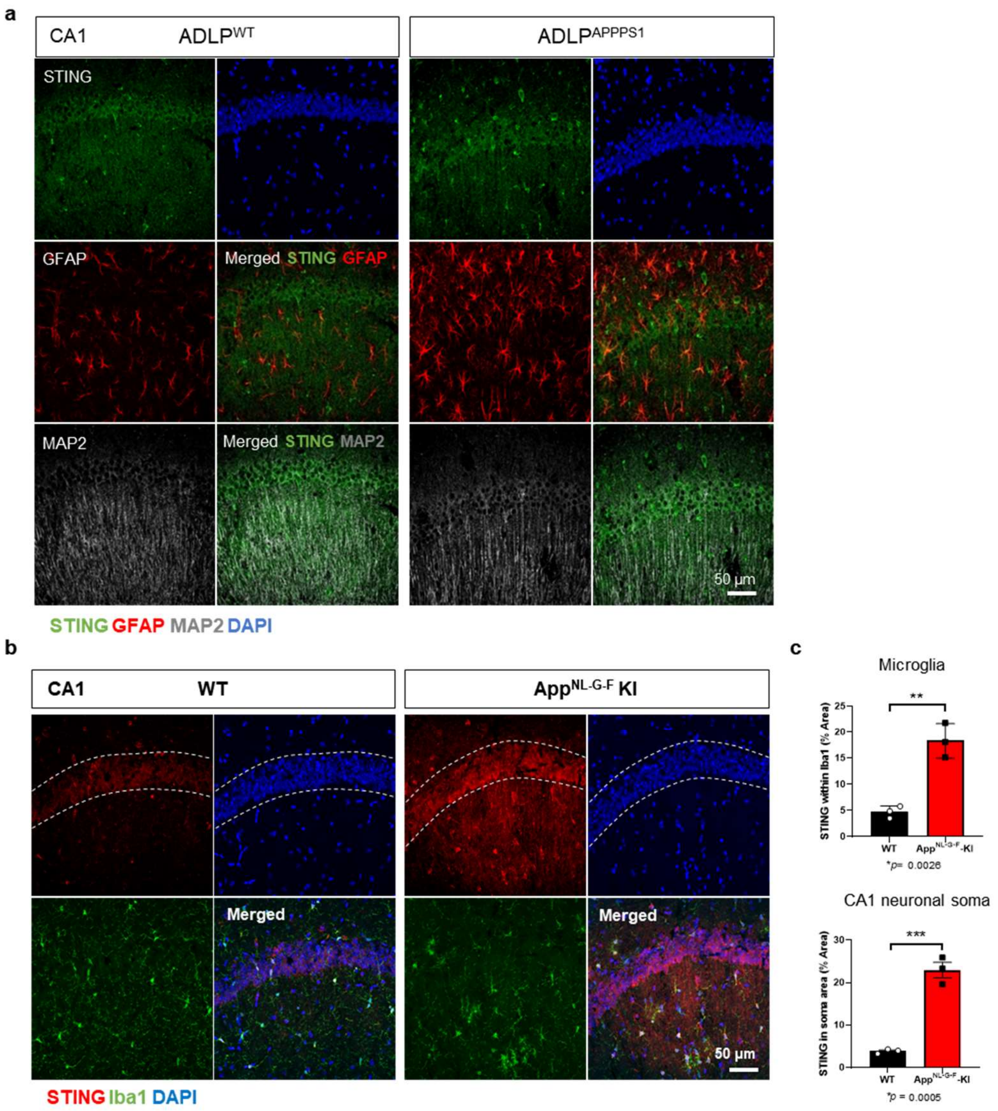

Supplementary Fig. 5. STING mainly overlaps with microglia and neurons in AD mice.

a, Representative immunostaining image of STING, GFAP, and MAP2 in WT and ADLP$^{APP/PS1}$ mice brain hippocampal CA1 region. Scale bars, 50 μm. **b**, Representative immunostaining of STING and Iba1 in WT and App$^{NL\text{-}G\text{-}F}$ mice brain. Scale bars, 50 μm. **c**, Quantification of STING positive signals in CA1 neuronal soma area and Iba1-masked microglia area. *N* =3,3.

In (**c**), Data are presented as mean ± s.e.m. Statistical significance was determined by Student's t-test. $^{**}p < 0.01$. $^{***}p < 0.001$. *N* indicates individual mice.

Supplementary Fig. 6

a

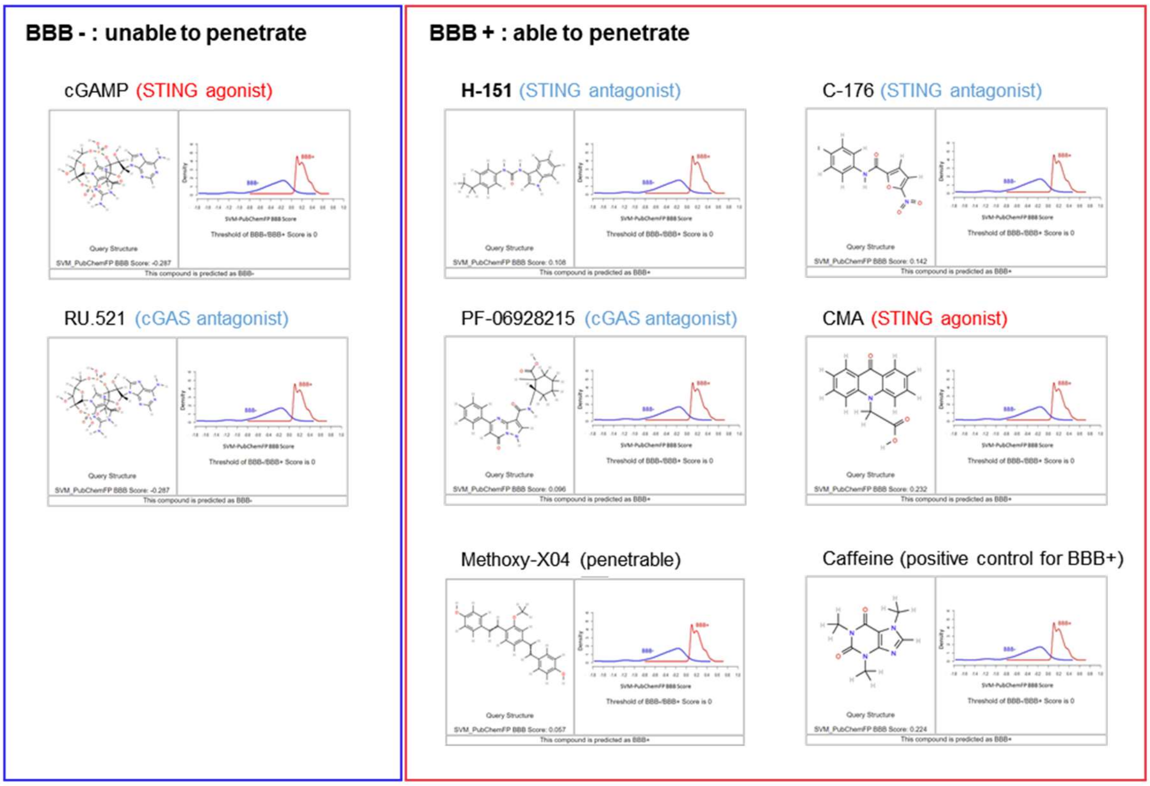

b

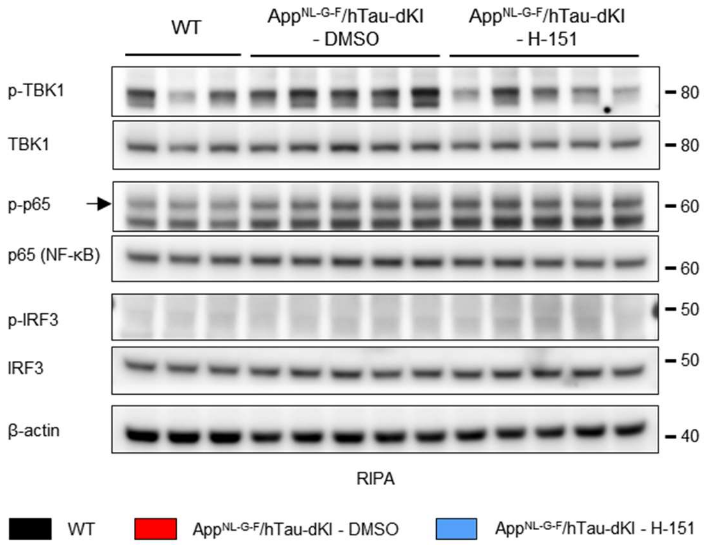

c

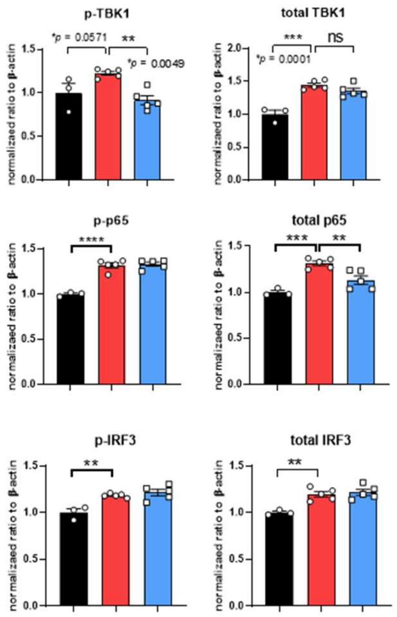

Supplementary Fig. 6. Blood Brain Barrier permeability predictive simulation for cGAS and STING agonist, antagonists with a confirmation for H-151.

a, Compound BBB permeability *in silico* prediction results of agonists and antagonists for cGAS and STING. Caffeine and methoxy-X04 were used as a BBB penetrable positive controls. **b**, Representative immunoblots for TBK1, NF-κB p65, IRF3 phosphorylation and total protein levels in H-151-administrated dKI mice brain. **c**, Quantification of protein levels in Suppl. Fig. 6b. N=3,5,5.

In (**c**), Data are presented as mean ± s.e.m. Statistical significance was determined by one-way ANOVA test with Tukey's multiple comparison test. $^{**}p < 0.01$. $^{***}p < 0.001$. N indicates individual mice.

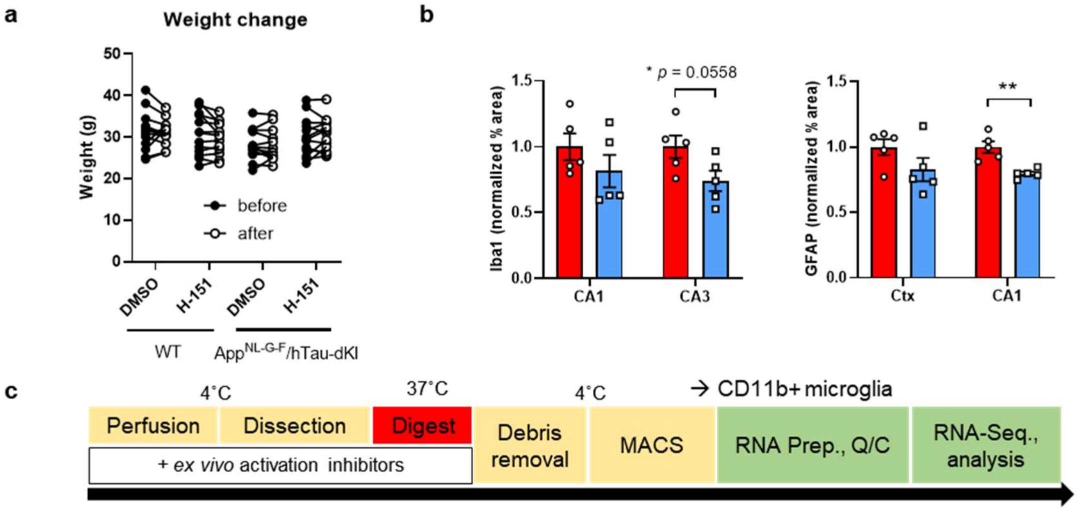

d

	Group1	Group2	Total DEGs	Up DEGs	Down DEGs
A	WT	A/T-dKI	769	397	372
B	WT DMSO	WT H-151	538	104	434
C	A/T-dKI DMSO	A/T-dKI H-151	166	34	132

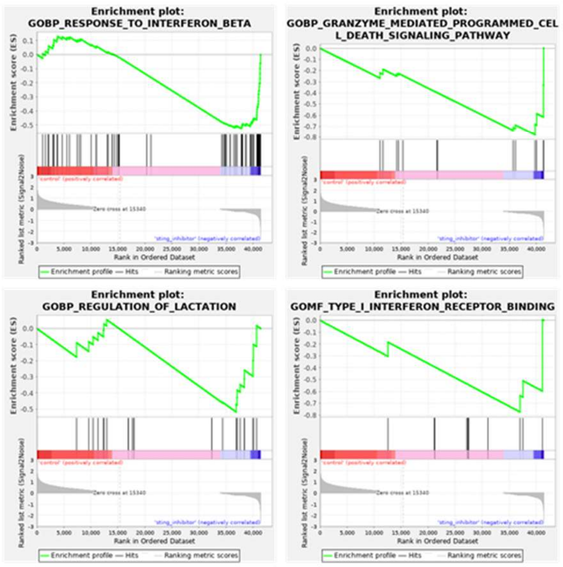

f

DMSO vs **H-151 in WT** and **dKI**

73 Shared **DOWN** DEGs

Rnf25, *Malat1*, *Bax*, *Psme2b*, *Gm49307*, *Hdac8*, *Ly9*, *Lamtor2*, *Gm47097*, *Bak1*, *Camk2n1*, *Kat5*, *Ambra1* ··· *Romo1*

Representative 14 DEGs

5 Shared **UP** DEGs

Xlr4c, *S100a8*, *Nme2*, *Spp1*, *Zfp984*

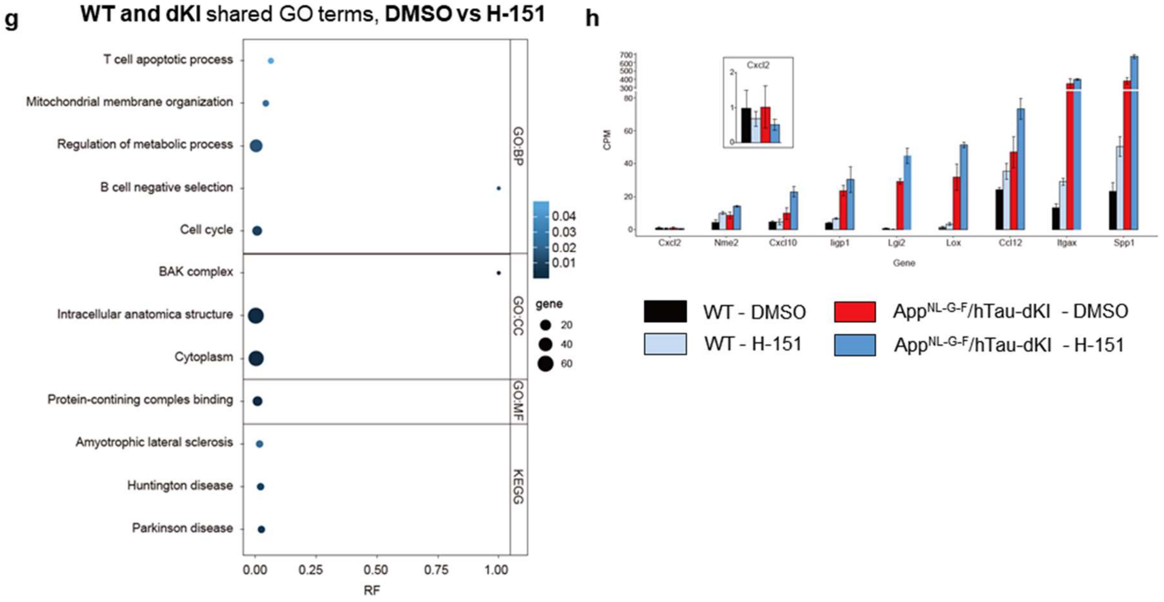

Supplementary Fig. 7. The effect of STING inhibition on microglial gene expression in App$^{NL\text{-}G\text{-}F}$/hTau-dKI mice.

a, Quantification of the weight change of mice before and after H-151 treatment. *N*=13 for each group. **b**, Quantification of Iba1 and GFAP %Area in hippocampus and cortex. *N*=5 for each group. **c**, Scheme of the MACS-based brain microglia isolation process used in the study. **d**, Microglial DEGs in each groups after applying the cutoff log2FC > |0.8|, p-value <0.05. **e**, GSEA analysis results of STING activity-dependent DEGs in dKI group. related with Fig. 5g. **f**, Common DEGs which shared in WT and dKI groups. Five upregulated DEGs and representative 14 DEGs from total 73 downregulated DEGs are listed. **g**, Highlighted GO terms of common 78 DEGs. **h**, STING-dependent upregulated DEGs CPM value. *N*=3 for each group.

In (**b**), Data are presented as mean ± s.e.m. Statistical significance was determined by two-tailed Student's t-test. $^{**}p < 0.01$. *N* indicates individual mice.

Supplementary Fig. 8

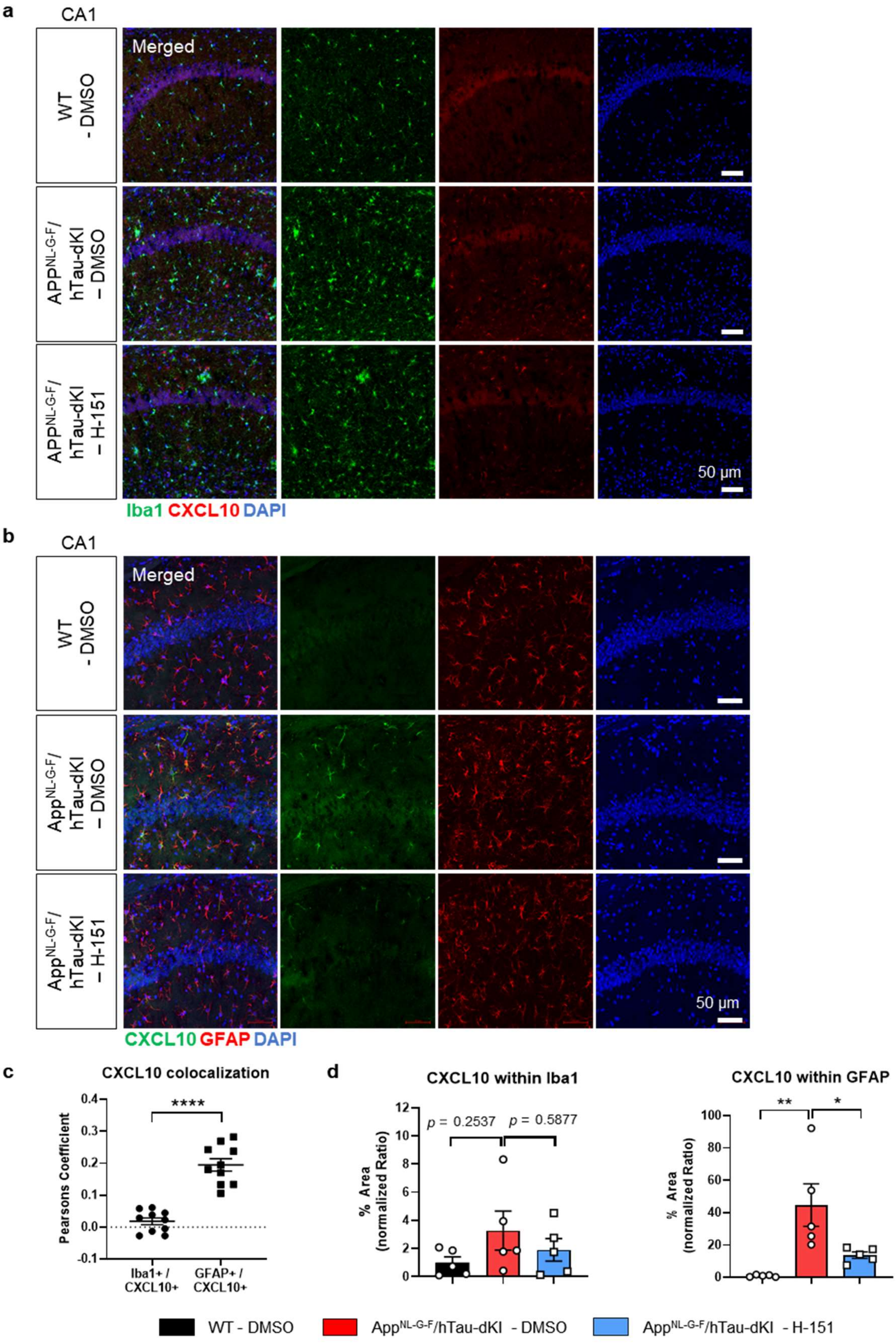

Supplementary Fig. 8. CXCL10 overlaps with astrocyte more than microglia.

a, Representative CXCL10 and Iba1 immunostaining images in WT and DMSO-, H-151-administrated $App^{NL\text{-}G\text{-}F}$/hTau-dKI mice. Scale bars, 50 μm. **b,** Representative CXCL10 and GFAP immunostaining images in identical groups. Scale bars, 50 μm. **c,** Pearson's correlation coefficient comparison result between CXCL10 with Iba1 and CXCL10 with GFAP. *N*=10,10. **d**, Quantification of CXCL10 immunostaining signal within Iba1, or GFAP positive area. *N*=5 for each group.

All data are presented as mean ± s.e.m. In (**b**), Statistical significance was determined by two-tailed Student's t-test. ****$p < 0.0001$. In (**c**), Statistical significance was determined by one-way ANOVA test with Tukey's multiple comparison test. *$p < 0.05$. **$p < 0.01$. *N* indicates individual mice.

Supplementary Fig. 9

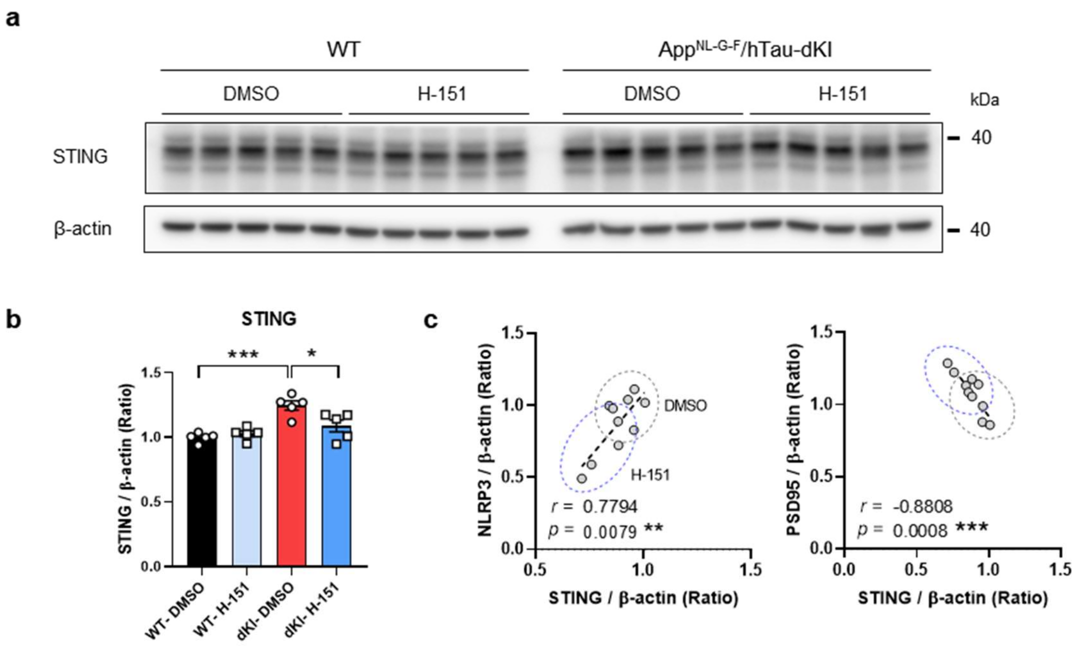

Supplementary Fig. 9. STING protein level change and correlation with NLRP3, PSD95.

a, Representative immunoblot of STING in DMSO-, H-151-injected WT and App^{NL-G-F}/hTau-dKI mice cerebral brain. **b**, Quantification of STING protein levels. *N*=5 for each group. **c**, Scatter plots for STING and NLRP3, PSD95 correlation. *N*=5 for each group.

In (**b**), Data are presented as mean ± s.e.m. Statistical significance was determined by two-way ANOVA test. **p* < 0.05. ****p* < 0.001. In (**c**), Data are presented as normalized ratio. Pearson's correlation *r* was used to present correlation strength and statistical significance was determined by two-tailed Student's t-test. ***p* < 0.01. ****p* < 0.001.

Author contributions

S.C. conducted overall experiments including biochemical, imaging, tissue analysis and behavioral studies, and wrote the manuscript; J.H.J. set up and contributed to behavioral tests and tissue analysis; J.C.P., J.W.H. contributed to human iMG differentiation and experiment. Y.L. and J.K. analyzed transcriptome data. I.M.-J. supervised the overall research, provided intellectual feedback, and wrote the manuscript.

1. Chung, S. *et al.* Plexin-A4 mediates amyloid-beta-induced tau pathology in Alzheimer's disease animal model. *Prog. Neurobiol.* **203**, 102075 (2021).

2. Liu, H. *et al.* AlzPlatform: an Alzheimer's disease domain-specific chemogenomics knowledgebase for polypharmacology and target identification research. *J. Chem. Inf. Model.* **54**, 1050-1060 (2014).
